# Homogenised balance equations for nematic liquid crystal flow in elastic porous media

**DOI:** 10.1007/s00033-025-02675-8

**Published:** 2026-02-20

**Authors:** Mohammed Alwady, Nigel J. Mottram, Raimondo Penta

**Affiliations:** 1 School of Mathematics and Statistics, University of Glasgow, Glasgow, G12 8QQ UK; 2 Department of Mathematics, Faculty of Science, P.O. Box 25145, Abha, 61466 Saudi Arabia

**Keywords:** Nematic liquid crystal, Asymptotic homogenisation, Fluid–Structure interaction, Porous media flow, Viscoelasticity, Poroelasticity, 76A15, 74F10, 74D05

## Abstract

We derive a new mathematical model for the macroscopic behaviour of a linear elastic porous medium weakly interacting with an incompressible, slowly flowing, nematic liquid crystal under the one elastic constant approximation and a simplified hypothesis concerning the fluid viscosities for which the stress tensor remains dependent on the nematic director, which is the average fluid molecular orientation, but is symmetric. In this situation, the angular momentum equation, which governs the dynamics of the nematic director, decouples from the linear momentum equations of the fluid. As such, whilst the nematic anisotropy affects the flow profile, the fluid flow no longer affects the configuration of the nematic director. We assume that the typical pore dimension (the *microscale*) is significantly smaller than the average size of the whole domain (the *macroscale*), and exploit this sharp length-scale separation in our use of the asymptotic homogenisation technique to derive new macroscale governing equations by upscaling the fluid–structure interaction problem between the porous elastic structure and the nematic liquid crystal fluid phase. The resulting novel anisotropic macroscale viscoelastic model describes the overall system representing a nematic liquid crystal flowing through an elastic porous solid and could therefore be termed an *anisotropic poro-viscoelastic model*. This system of partial differential equations incorporates the nematic director, its spatial variations, and the underlying microstructure through coefficients that are computed by solving appropriate microscale cell problems. The homogenised constitutive relationships account for the roles of the nematic director field, the elastic response of the solid phase, and their interplay with the underlying microstructural configuration. We then focus on the particular case in which the role of the elastic porous structure is negligible. In this case, the fluid flow is no longer affected by the deformations of the porous medium and is solely driven by a volume load, which depends on macroscale spatial variations of the nematic director and its interplay with the underlying microscale geometry. The resulting theoretical framework opens up new modelling possibilities for a wide range of potential applications for nematic liquid crystals encapsulated in a complex porous network.

## Introduction

Real-world physical systems are often intrinsically multiscale, strongly heterogeneous, geometrically complex, and characterised by multiple constituents that can interact through several hierarchical levels of organisation, for instance in systems where fluid flows through non-trivial porous structures featuring mechanical and chemical interactions. A major challenge resides in providing a comprehensive representation of such systems, which accounts for such interactions, given that it is normally challenging, and sometimes practically impossible, to resolve the fine details of the microstructure, both from an experimental and a computational modelling point of view. A convenient approach to tackle these problems consists of providing a coarser scale description of the overall behaviour of such systems via appropriate *homogenisation* techniques, such as mixture theory, as described in the review [61], or asymptotic homogenisation, see, e.g. the book [34]. While mixture theory approaches have proven very useful in the study of the behaviour of porous media flow and model closure, in this work we focus on the *asymptotic homogenisation technique* since we aim to provide a coarse scale representation for porous media flow which is capable of featuring an explicit link between the pore structure at the *microscale* and the overall behaviour at the *macroscale*. Relevant examples of applications of the asymptotic homogenisation techniques to upscale fluid–structure interaction problems in porous media can be found for both Newtonian and non-Newtonian fluids, e.g. [65] and [13], respectively, and for rigid, deformable, and evolving porous media, see, e.g. [44, 51, 52], and [57], respectively.

A nematic liquid crystal is an example of a non-Newtonian fluid, which consists of rod-like (or disc-like) organic molecules and, in a temperature range between the classical liquid and crystalline solid phases, forms a phase in which the molecules retain orientational order without positional order. This orientational order derives from a tendency for the molecules to align themselves along a preferred direction, referred to as the director, while maintaining the ability to flow like a fluid [17, 24, 48]. The interaction between molecular orientation and flow can lead to complex behaviour, which has been the focus of many studies over the past few decades [18, 38]. The orientational order of these materials allows the persistence of an internal molecular structure, stored elasticity, molecular reorientation upon the application of an electric field, and an ability to direct the polarisation of incident light, all of which is used to create pixelated electro-optic devices—the now ubiquitous Liquid Crystal Display [19].

There have been a number of studies devoted to liquid crystals confined in various forms of porous media, for instance in high-precision porous membranes, porous glass, aerosols, and aerogels [22, 40]. Early studies focussed on the effect of the porous medium on orientational ordering, and phase behaviour, where the porous medium was found to influence the local orientational ordering, the coherence length of the ordering and the transition behaviour between phases [9, 22, 23, 28–30, 32, 35, 41, 58–60, 71, 74]. More recent studies have considered the absorption of a liquid crystal into a porous medium [36], memory and defect stabilisation effects of the porous medium [3–5, 62], and the possible use of liquid crystal–porous medium systems for optical and electro-optical devices [26, 27, 39, 66, 67, 70, 72].

Theoretical investigations of liquid crystals within porous media are often based on a simplified, sometimes unconnected, form of the pores [8, 20] or on an approach that uses a generalised mean field to model random or ordering effects of the porous medium [22, 41]. Those models which have used a more detailed consideration of the pore characteristics, for instance by using homogenisation techniques similar to those in this paper, have largely been concerned with the inclusion of static particles [10, 11, 15, 16] or anisotropic diffusion properties [63]. However, the study of the flow of anisotropic liquids within a porous medium has not received sufficient attention so far, despite the existing technological importance of a specific class of anisotropic liquids, namely nematic liquid crystals, in which flow can be important in both the production and operation of devices [21, 73]. Although current nematic liquid crystal technologies focus on very simple pore structures, such as a single thin layer [64] or a highly connected, low-weight polymer matrix [47], the potential applications of more complicated porous media, for instance as elastically stabilising environments, mean that a rigorous model of such structures would be extremely valuable.

The objective of this work is to develop a homogenised system of partial differential equations that describes the behaviour of a nematic liquid crystal interacting with a solid porous structure whilst retaining a clear link between the underlying microstructure and the macroscale response, under a number of simplifying assumptions.

In Section 2, we begin by formulating the governing equations by treating the solid phase as a linear elastic pore structure under the assumption of negligible inertia and no body forces, while assuming that the fluid is an incompressible viscous nematic liquid crystal. We consider a simplified formulation of the Ericksen–Leslie constitutive relationship for the nematic fluid flow (see, e.g. [69]) such that the resulting fluid stress tensor is now symmetric, but retains a dependence on the director orientation. With this approximation there is no influence of the fluid flow on the nematic director, although the director does influence the flow. Interface conditions are also needed to close the system, and we impose continuity of velocities and tractions across the fluid-solid boundary. We non-dimensionalise these equations in Section 3 to consider the relative importance of the various physical phenomena. In Section 4, we then introduce the asymptotic homogenisation technique (see, e.g. [6, 33, 43, 49, 65]), expressing the relevant fields for the fluid phase (velocity, nematic director, Lagrange multiplier related to the unit norm constraint, pressure, and stress tensor) and the solid phase (the displacement and the stress tensor) as power series in the scale separation parameter $\epsilon$, which is the ratio between the representative microscopic and macroscopic length scales. In Section 5, we apply the asymptotic homogenisation method to derive a new set of macroscale governing equations. This process includes decoupling the spatial variations and employing periodicity assumptions to close the system that describes the leading-order macroscopic behaviour of the velocity, director, and pressure fields, as well as the behaviour of the elastic displacement. In Section 6, we summarise and discuss the main results, then, in 6.1, illustrate a simplified formulation of our new model by considering the case in which the contribution of the solid stresses to the macroscale linear momentum balance is negligible. In Section 7 we conclude by discussing major novelties, limitations, and directions for future work.

## Formulation of the fluid–structure interaction problem

**Fig. 1:**
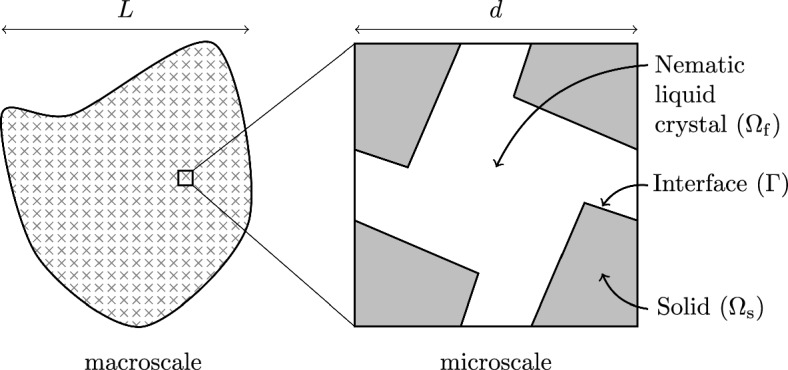
A schematic of a 2D cross-section of the macroscale region (left) and a single periodic microscale region (right). In the microscale, the fluid domain is denoted in white and the solid domain in grey

We consider a fluid–structure interaction between a porous solid phase and an incompressible nematic liquid crystal slowly flowing through the pores. We represent the whole domain as a set $$\tilde{\Omega } \subseteq {\mathbb {R}}^n$$ with $$ n=2$$ or 3, such that $$\bar{\tilde{\Omega }}=\bar{\tilde{\Omega }}_{\textrm{f}} \cup \bar{\tilde{\Omega }}_{\textrm{s}}$$, where $$\tilde{\Omega }_{\textrm{f}}$$ and $$\tilde{\Omega }_{\textrm{s}}$$ stand for the fluid and solid regions, respectively, and the overline symbol indicates that the boundaries in each domain are included. We remark that the model presented here applies to both two- and three-dimensional scenarios; in particular, we have chosen to exemplify the geometrical set-up of our model via a schematic of a 2D cross section of the domain for the sake of illustrative purposes, see Figure 1. Before presenting the governing equation of each domain within our system, we provide a clarification of the notation employed throughout this manuscript.

### Remark 1

(Notation) Scalars are written as ordinary lowercase letters, e.g. *v*, with vectors denoted by bold lowercase letters, e.g. $\boldsymbol{v}$. An uppercase sans-serif font $\textsf{V}$ is used to represent second-rank tensors. For third-rank tensors, we utilise uppercase calligraphic letters, like $\mathcal {V}$, while we employ blackboard font ${\mathbb {V}}$ for fourth-rank tensors. Fifth-rank tensors are represented by bold sans-serif  $\boldsymbol{\textsf{V}}$, and sixth-rank tensors are denoted using monospaced font, such as $\texttt{V}$.

We assume that the typical length scale of pores *d* is much smaller than the average size of the whole domain *L*. Therefore, the ratio between those length scales can be defined as a small parameter as

1 $$\begin{aligned} \epsilon =\frac{d}{L} \ll 1, \end{aligned}$$

which therefore measures the spatial scale separation between the microscale *d* and the macroscale *L*. A depiction of the porous microstructure is presented in Fig. 1. We now state the governing equations for the porous solid and fluid components, where the fluid is assumed to be a nematic liquid crystal.

The nematic liquid crystal is assumed to be an incompressible viscous fluid [24] with no external forces, and we consider low Reynolds number flow. We can therefore describe the fluid dynamics by means of the following linear momentum equation and incompressibility constraint,

2a $$\begin{aligned} \nabla \cdot \mathsf {\sigma }_{\textrm{f}}&= 0, \end{aligned}$$

2b $$\begin{aligned} \nabla \cdot \boldsymbol{v}&= 0, \end{aligned}$$

where $\mathsf {\sigma }_{\textrm{f}}$ denotes the fluid stress tensor and $\boldsymbol{v}$ is the fluid velocity. Due to the orientational order within a nematic liquid crystal, which defines the local average molecular orientation, the director $\boldsymbol{m}(\boldsymbol{x},t)$, we must also consider the balance of angular momentum [69]. Coupling between the linear and angular momentum occurs through the viscous stress tensor, which, in the case of a nematic, depends on both velocity gradients and the director orientation. This coupling means that the director orientation, distortions, and dynamics will influence the flow profile, and velocity gradients will affect the director configuration. However, in this paper, in order to concentrate on the influence of the director on the macroscopic homogenised equations for flow in the porous medium, we study a simplified version of the Ericksen–Leslie constitutive relationship for nematic liquid crystals, wherein the viscous stress tensor is assumed to be symmetric while maintaining its dependency on the director orientation. Specifically, among the six Leslie viscosity coefficients $\alpha _i,\ i=1\ldots 6$, we neglect $\alpha _2$ and $\alpha _3$. Assuming the standard Parodi relation, see, e.g. [69], this also implies that $\alpha _5=\alpha _6{:}{=}\alpha$ and so the viscous stress contains only three independent viscosities. This assumption results in a symmetric viscous stress tensor and a partial decoupling of the director and the flow field. Specifically, and physically, this means that although the nematic director affects the fluid flow, through the nonlinear dependence of the effective viscosity on the director, the director is influenced only by the elastic behaviour and boundary interactions, and not by the fluid flow. In particular, both the so-called *rotational viscosity*, $\gamma _1=\alpha _2-\alpha _3$, and the *torsion coefficient*, $\gamma _2=\alpha _2+\alpha _3$, are zero in our model, which removes the flow–alignment (or tumbling) response of the nematic director to shear, see [69]. The role of these viscosities will become less important in situations where the elastic and boundary effects dominate, such as in highly confined regions and when the temperature of the material is close to the point at which the liquid crystal reverts to an isotropic liquid [50].

With the above assumption, the most general constitutive equation for a nematic liquid crystal is then given by

3 $$\begin{aligned} \mathsf {\sigma }_{\textrm{f}}=-p\textsf{I}-2K (\nabla \boldsymbol{m})^T(\nabla \boldsymbol{m})+\alpha _{1}(\boldsymbol{m} \cdot \textsf{D}\left( \boldsymbol{v}\right) \boldsymbol{m}) \boldsymbol{m} \otimes \boldsymbol{m}+ 2\mu \textsf{D}(\boldsymbol{v})+\alpha \bigg (\textsf{D}\left( \boldsymbol{v}\right) \boldsymbol{m} \otimes \boldsymbol{m}+ \boldsymbol{m} \otimes \textsf{D}\left( \boldsymbol{v}\right) \boldsymbol{m}\bigg ),\nonumber \\ \end{aligned}$$

where *p* is the pressure, $\textsf{I}$ is the identity tensor, *K* is the one-constant Frank elasticity quantifying the material resistance to distortions in the director field [69], $\boldsymbol{m}(\boldsymbol{x})$ is the nematic director, $\textsf{D}(\boldsymbol{v})=\left( \nabla \boldsymbol{v}+(\nabla \boldsymbol{v})^T\right) /2$ is the symmetric rate of strain tensor, $\mu$ is the Newtonian viscosity (which is equal to $\alpha _4/2$ in terms of the relevant Leslie viscosity) and $\alpha _1,\,\alpha$ are the remaining Leslie viscosities mentioned above.

We describe the mechanical response of the solid phase as that of a linear elastic material, such that by neglecting inertia and body forces, we have

4 $$\begin{aligned} \nabla \cdot \mathsf {\sigma }_{\textrm{s}}&=0 \quad \text{ in } \ \tilde{\Omega }_{\textrm{s}}, \end{aligned}$$

where $\mathsf {\sigma }_{\textrm{s}}$ is the solid stress tensor which is given by

5 $$\begin{aligned} \mathsf {\sigma }_{\textrm{s}}= {\mathbb {C}}: \nabla \boldsymbol{u}. \end{aligned}$$

Here, $\boldsymbol{u}$ is the displacement vector for the solid phase and ${\mathbb {C}}$ is the fourth-rank elasticity tensor, which can be written in components as $C_{ijkl}$, for $i,j, k,l\in \{1,\ldots ,n\}$. The operation “ : ” that appears in (5) is the standard double contraction between tensors of rank two or higher, for instance relationship (5) reads, component-wise,

6 $$\begin{aligned} C_{ijkl}\frac{\partial {u_k}}{\partial x_l}, \end{aligned}$$

where summation over repeated indices is understood, here and throughout this work, unless otherwise specified. The elasticity tensor possesses right minor and major symmetries, specifically

7a $$\begin{aligned} C_{ijkl}= C_{ijlk}, \end{aligned}$$

7b $$\begin{aligned} C_{ijkl}= C_{klij}, \end{aligned}$$

and therefore by combining (7a–7b), we also obtain left minor symmetries. More precisely, by applying right minor symmetries, we can reformulate constitutive equation (5) equivalently as

8 $$\begin{aligned} \mathsf {\sigma }_{\textrm{s}}= {\mathbb {C}}: \textsf{D}(\boldsymbol{u}), \end{aligned}$$

where $\textsf{D}(\boldsymbol{u})=\left( \nabla \boldsymbol{u}+(\nabla \boldsymbol{u})^T\right) /2$ is the linearised strain.

In order to close the system of partial differential equations (PDEs) describing the fluid–structure interaction problem, we require appropriate interface conditions on the interface between the fluid and solid phases, denoted by $\tilde{\Gamma }=\partial \tilde{\Omega }_{\textrm{f}} \cap \partial \tilde{\Omega }_{\textrm{s}}$. In particular, we assume that velocities and tractions across the interface are continuous, that is

9a $$\begin{aligned} \dot{\boldsymbol{u}}&= \boldsymbol{v} \quad \text {on }\tilde{ \Gamma }, \end{aligned}$$

9b $$\begin{aligned} \mathsf {\sigma }_{\textrm{f}} \boldsymbol{n}&= \mathsf {\sigma }_{\textrm{s}} \boldsymbol{n} \quad \text {on } \tilde{ \Gamma }. \end{aligned}$$

where $\boldsymbol{n}$ is the unit outward vector normal to the interface $\tilde{\Gamma }$.

## Non-dimensional analysis

In this section, we non-dimensionalise the system of PDEs in order to understand the correct asymptotic behaviour of the relevant fields at hand, with respect to the scale separation parameter $\epsilon$. We assume that the system is defined by a characteristic reference velocity, denoted by *V* and a characteristic pressure gradient, denoted by *C*, along with the relevant length scales to obtain dimensionless quantities as follows:

10 $$\begin{aligned}  &   \boldsymbol{x}=L \boldsymbol{x}^{\prime },\ \boldsymbol{u}=L\boldsymbol{u}^{\prime },\ p=C L p^{\prime }, \ \boldsymbol{v}=V \boldsymbol{v}^{\prime },\nonumber \\  &   \nabla =\dfrac{1}{L} \nabla ^{\prime },\ \mathsf {\sigma }_\textrm{f}=C L \mathsf {\sigma }_\textrm{f}^{\prime },\ \mathsf {\sigma }_\textrm{s}=C L \mathsf {\sigma }_\textrm{s}^{\prime },\nonumber \\  &   {\mathbb {C}}= CL {\mathbb {C}}^{\prime },\ \textsf{D}(\boldsymbol{v})=\dfrac{V}{L} \textsf{D}^{\prime }\left( \boldsymbol{v}^{\prime }\right) =\dfrac{V}{L}\left( \dfrac{\nabla ^{\prime } \boldsymbol{v}^{\prime }+\left( \nabla ^{\prime } \boldsymbol{v}^{\prime }\right) ^T}{2}\right) . \end{aligned}$$

Selecting different scalings for the fluid velocity and pressure allows the effective behaviour of different porous media flows to be captured, as in [44, 54].

Neglecting the primes for the sake of simplicity of notation yields the following non-dimensional system of PDEs:

11a $$\begin{aligned} \displaystyle \nabla \cdot \mathsf {\sigma }_\textrm{f} = 0&\qquad \qquad \text{ in } \ \tilde{\Omega }_{\textrm{f}}, \end{aligned}$$

11b $$\begin{aligned} \displaystyle \nabla \cdot \boldsymbol{v} =0&\qquad \qquad \text{ in } \ \tilde{\Omega }_{\textrm{f}},\end{aligned}$$

11c $$\begin{aligned} \displaystyle \nabla \cdot \mathsf {\sigma }_\textrm{s} = 0&\qquad \qquad \text{ in } \ \tilde{\Omega }_{\textrm{s}}, \end{aligned}$$

11d $$\begin{aligned} \displaystyle -\nabla ^2 \boldsymbol{m}+\lambda \boldsymbol{m} = 0&\qquad \qquad \text{ in } \ \tilde{\Omega }_{\textrm{f}},\end{aligned}$$

11e $$\begin{aligned} \displaystyle \Vert \boldsymbol{m}\Vert ^{2} = 1&\qquad \qquad \text{ in } \ \tilde{\Omega }_{\textrm{f}},\end{aligned}$$

11f $$\begin{aligned} \displaystyle \boldsymbol{v} = \dot{\boldsymbol{u}}&\qquad \qquad \text{ on } \ \tilde{\Gamma }, \end{aligned}$$

11g $$\begin{aligned} \displaystyle \mathsf {\sigma }_{\textrm{f}} \boldsymbol{n} = \mathsf {\sigma }_{\textrm{s}}\boldsymbol{n}&\qquad \qquad \text{ on } \ \tilde{\Gamma }, \end{aligned}$$

11h $$\begin{aligned} \displaystyle (\nabla \boldsymbol{m}) \boldsymbol{n} = 0&\qquad \qquad \text{ on } \ \tilde{\Gamma }, \end{aligned}$$

11i $$\begin{aligned} \displaystyle \mathsf {\sigma }_\textrm{f} = -p\textsf{I}-\bar{K}(\nabla \boldsymbol{m})^T(\nabla \boldsymbol{m})+ 2\bar{\mu } \textsf{D}(\boldsymbol{v})\qquad&\qquad \qquad \nonumber \\ +2\bar{\mu } \bar{\alpha }_{1}(\boldsymbol{m}\cdot \textsf{D}\left( \boldsymbol{v}\right) \boldsymbol{m} ) \boldsymbol{m} \otimes \boldsymbol{m} \displaystyle + 2 \bar{\mu } \bar{\alpha }\bigg ( \textsf{D} (\boldsymbol{v}) \boldsymbol{m} \otimes \boldsymbol{m}+\boldsymbol{m} \otimes \textsf{D}(\boldsymbol{v}) \boldsymbol{m}\bigg )&\qquad \qquad \text{ in } \ \tilde{\Omega }_{\textrm{f}}, \end{aligned}$$

11j $$\begin{aligned} \displaystyle \mathsf {\sigma }_{\textrm{s}} = {\mathbb {C}} :\textsf{D} (\boldsymbol{u})&\qquad \qquad \text{ in } \ \tilde{\Omega }_{\textrm{s}}, \end{aligned}$$

with the non-dimensional numbers defined as

12 $$\begin{aligned} \begin{aligned} \bar{K}=\frac{2K}{C L^3 }, \quad \bar{\mu }=\frac{V \mu }{C L^2},\quad \bar{\alpha }=\frac{\alpha }{2\mu },\quad \bar{\alpha }_{1}=\frac{\alpha _{1}}{2\mu }. \end{aligned}, \end{aligned}$$

and $\lambda$ is the Lagrange multiplier associated with constraint (11e).

## Two-scale asymptotic homogenisation

We now apply the asymptotic homogenisation technique [2, 6, 7, 12, 54] to derive a new set of macroscale governing equations for the system of PDEs (11a–11j). In the light of the sharp length-scale separation between the microscale and the macroscale, i.e.

13 $$\begin{aligned} \epsilon \ll 1, \end{aligned}$$

we introduce a new spatial variable

14 $$\begin{aligned} \boldsymbol{y}=\frac{\boldsymbol{x}}{\epsilon }, \end{aligned}$$

to consider microscopic variations of the fields. We decouple spatial variations so that $\boldsymbol{x}$ and $\boldsymbol{y}$ are considered formally independent variables that account for macroscale and microscale spatial variations, respectively. As a consequence, differential operators transform according to

15 $$\begin{aligned} \nabla \rightarrow \nabla _{\boldsymbol{x}}+\frac{1}{\epsilon } \nabla _{\boldsymbol{y}}. \end{aligned}$$

We represent each relevant field (collectively denoted as $\hat{f}$) through the following expansion in power series of $\epsilon$

16 $$\begin{aligned} \hat{f} \equiv \hat{f}^\epsilon (\boldsymbol{x}, \boldsymbol{y})=\sum _{l=0}^{\infty } \hat{f}^{(l)}(\boldsymbol{x}, \boldsymbol{y}) \epsilon ^l. \end{aligned}$$

Following a commonly exploited asymptotic homogenisation approach (see, e.g.[7, 12, 65] amongst many others), we assume that every field is periodic with respect to the microscopic variable $\boldsymbol{y}$. As such, it is sufficient to study fine scale variations of the fields on the single periodic cell, which we denote by $\Omega$. We further identify $\Omega _{\textrm{f}}$ and $\Omega _{\textrm{s}}$ with their corresponding portions of the periodic cell, and $\Gamma$ with their interface, see Figure 1.

Exploiting the representation (16) and the spatial decoupling (15), Eqs. (11a–11j) read

17a $$\begin{aligned} \displaystyle \nabla _{\boldsymbol{y}} \cdot \mathsf {\sigma }_\textrm{f}^\epsilon +\epsilon \nabla _{\boldsymbol{x}} \cdot \mathsf {\sigma }_\textrm{f}^\epsilon&=0 \quad  &   \text{ in } \ \Omega _{\textrm{f}}, \end{aligned}$$

17b $$\begin{aligned} \displaystyle \nabla _{\boldsymbol{y}} \cdot \boldsymbol{v}^\epsilon +\epsilon \nabla _{\boldsymbol{x}} \cdot \boldsymbol{v}^\epsilon&=0 \    &   \text{ in } \ \Omega _{\textrm{f}},\end{aligned}$$

17c $$\begin{aligned} \displaystyle \nabla _{\boldsymbol{y}} \cdot \mathsf {\sigma }_\textrm{s}^\epsilon +\epsilon \nabla _{\boldsymbol{x}} \cdot \mathsf {\sigma }_\textrm{s}^\epsilon&=0 \quad  &   \text{ in } \ \Omega _{\textrm{s}}, \end{aligned}$$

17d $$\begin{aligned} \displaystyle -\epsilon ^2 \nabla _{\boldsymbol{x}}^2 \boldsymbol{m}^{\epsilon } - \nabla _{\boldsymbol{y}}^2 \boldsymbol{m}^{\epsilon } - \epsilon \nabla _{\boldsymbol{y}} \cdot \nabla _{\boldsymbol{x}} \boldsymbol{m}^{\epsilon } - \epsilon \nabla _{\boldsymbol{x}} \cdot \nabla _{\boldsymbol{y}} \boldsymbol{m}^{\epsilon } +\epsilon ^{2} \lambda ^{\epsilon } \boldsymbol{m}^{\epsilon }&=0 \quad  &   \text{ in } \ {\Omega }_{\textrm{f}},\end{aligned}$$

17e $$\begin{aligned} \displaystyle \Vert \boldsymbol{m}^{\epsilon }\Vert ^{2}&=1 \quad  &   \text{ in } \ {\Omega }_{\textrm{f}}, \end{aligned}$$

17f $$\begin{aligned} \displaystyle \boldsymbol{v}^\epsilon&=\dot{\boldsymbol{u}}^\epsilon \quad  &   \text{ on } \ \mathrm {\Gamma },\end{aligned}$$

17g $$\begin{aligned} \displaystyle \mathsf {\sigma }^{\epsilon }_{\textrm{f}} \boldsymbol{n}&=\mathsf {\sigma }^{\epsilon }_{\textrm{s}} \boldsymbol{n} \quad  &   \text{ on } \ \Gamma ,\end{aligned}$$

17h $$\begin{aligned} \displaystyle \epsilon \left( \nabla _{\boldsymbol{x}} \boldsymbol{m}^{\epsilon } \right) \boldsymbol{n} + \left( \nabla _{\boldsymbol{y}} \boldsymbol{m}^{\epsilon } \right) \boldsymbol{n}&=0 \quad  &   \text{ on } \ {\Gamma }, \end{aligned}$$

17i $$\begin{aligned} \displaystyle \epsilon ^2 \mathsf {\sigma }_\textrm{f}^\epsilon = - \epsilon ^2 p^\epsilon \textsf{I}+ 2\bar{\mu } \left( \epsilon \textsf{D}_{\boldsymbol{y}}\left( \boldsymbol{v}^\epsilon \right) +\epsilon ^2 \textsf{D}_{\boldsymbol{x}} \left( \boldsymbol{v}^\epsilon \right) \right)&\nonumber \\ \displaystyle - \bar{K}\left( (\nabla _{\boldsymbol{y}} \boldsymbol{m}^\epsilon )^{\top } \nabla _{\boldsymbol{y}} \boldsymbol{m}^\epsilon +\epsilon (\nabla _{\boldsymbol{x}} \boldsymbol{m}^\epsilon )^{\top }\nabla _{\boldsymbol{y}}\boldsymbol{m}^\epsilon +\epsilon (\nabla _{\boldsymbol{y}} \boldsymbol{m}^\epsilon )^{\top } \nabla _{\boldsymbol{x}} \boldsymbol{m}^\epsilon +\epsilon ^2 (\nabla _{\boldsymbol{x}} \boldsymbol{m}^\epsilon )^{\top }\nabla _{\boldsymbol{x}}\boldsymbol{m}^\epsilon \right)&\nonumber \\ \displaystyle +\big (\boldsymbol{m}^\epsilon \otimes 2 \bar{\alpha }\bar{\mu }\epsilon \textsf{D}_{\boldsymbol{y}} (\boldsymbol{v}^\epsilon ) \boldsymbol{m}^\epsilon \big )+\big ( \boldsymbol{m}^\epsilon \otimes 2 \bar{\alpha }\bar{\mu } \epsilon ^2 \textsf{D}_ {\boldsymbol{x}} (\boldsymbol{v}^\epsilon ) \boldsymbol{m}^\epsilon \big )&\nonumber \\ \displaystyle +\left( 2\bar{\alpha }\bar{\mu } \epsilon \textsf{D}_{\boldsymbol{y}} (\boldsymbol{v}^\epsilon ) \boldsymbol{m}^\epsilon \otimes \boldsymbol{m}^\epsilon \right) +\big (2 \bar{\mu } \bar{\alpha }_{1}(\boldsymbol{m}^\epsilon \cdot \epsilon \textsf{D}_{\boldsymbol{y}}\left( \boldsymbol{v}^\epsilon \right) \boldsymbol{m}^\epsilon ) \boldsymbol{m}^\epsilon \otimes \boldsymbol{m}^\epsilon \big )&\nonumber \\ \displaystyle +\big (2\bar{\mu } \bar{\alpha }_{1} (\boldsymbol{m}^\epsilon \cdot \epsilon ^2\textsf{D}_{\boldsymbol{x}}\left( \boldsymbol{v}^\epsilon \right) \boldsymbol{m}^\epsilon ) \boldsymbol{m}^\epsilon \otimes \boldsymbol{m}^\epsilon \big )+\big (2\bar{\alpha }\bar{\mu } \epsilon ^2 \textsf{D}_{\boldsymbol{x}} (\boldsymbol{v}^\epsilon ) \boldsymbol{m}^\epsilon \otimes \boldsymbol{m}^\epsilon \big )&\qquad \qquad \qquad \text{ in } \ {\Omega }_{\textrm{f}}, \end{aligned}$$

17j $$\begin{aligned} \qquad \qquad \qquad \qquad \qquad \qquad \qquad \qquad \qquad \qquad \qquad \displaystyle \epsilon \mathsf {\sigma }_\textrm{s}^\epsilon = {\mathbb {C}}: \textsf{D}_{\boldsymbol{y}} (\boldsymbol{u}^\epsilon ) + \epsilon {\mathbb {C}}: \textsf{D}_{\boldsymbol{x}} (\boldsymbol{u}^\epsilon )&\qquad \qquad \qquad \text{ in } \ {\Omega }_{\textrm{s}}, \end{aligned}$$

where the operators $\textsf{D}_{\boldsymbol{x}}$ and $\textsf{D}_{\boldsymbol{y}}$ represent the symmetric part of the gradient operator with respect to the macroscale $\boldsymbol{x}$ and microscale $\boldsymbol{y}$, respectively, and the fields $\mathsf {\sigma }^\epsilon$, $\boldsymbol{v}^\epsilon$, $p^\epsilon$, $\boldsymbol{m}^\epsilon$, $\boldsymbol{u}^{\epsilon }$, $\lambda ^{\epsilon }$ are the power series counterparts [cf. Eq. (16)] of $\mathsf {\sigma }$, $\boldsymbol{v}$, *p*, $\boldsymbol{m}$, $\boldsymbol{u}$, $\lambda$, respectively. Since we aim to obtain a system of PDEs that holds on the macroscale only, it is useful to define the following cell average operator for any field $\hat{f}$ as

18 $$\begin{aligned} \langle \hat{f}\rangle _{\textrm{r}}=\frac{1}{|\Omega |} \int _{\Omega _\textrm{r}} \hat{f}( \boldsymbol{x}, \boldsymbol{y}) \hspace{2mm} \textrm{d} \boldsymbol{y} \quad \textrm{r}=\textrm{f},\textrm{s}, \end{aligned}$$

where $|\Omega |$ is the volume of the periodic cell and we have denoted the solid and fluid portions by $\Omega _{\textrm{s}}$ and $\Omega _{\textrm{f}}$, respectively.

### Remark 2

(Macroscopic uniformity) As is standard, we assume macroscopic uniformity by neglecting the geometrical variations of the microscale with respect to the macroscale $\boldsymbol{x}$ for the sake of simplicity. This assumption allows us to limit our analysis to a single periodic cell for every macroscale point $\boldsymbol{x}$. This means that we assume $\Omega =\Omega ( \boldsymbol{y})$ so that the following differentiation under the integral sign holds

19 $$\begin{aligned} \left\langle \nabla _{ \boldsymbol{x}} \cdot (\bullet )\right\rangle _{\textrm{r}}=\nabla _{ \boldsymbol{x}} \cdot \langle (\bullet )\rangle _{\textrm{r}} \quad \textrm{r}=\textrm{f},\textrm{s}, \end{aligned}$$

where $\langle (\bullet )\rangle _{\textrm{r}}$ denotes the average in Eq. (18).

### Remark 3

(Weak fluid–structure interaction) We ignore the contributions due to higher-order solid velocities, that is

$$ \dot{\boldsymbol{u}}^{(l)}=0,\ \ \text {for}\ \ l=1,2,\ldots ,$$

so that in particular $\dot{\boldsymbol{u}}^{(1)}=0$. Since higher-order solid displacements are related to spatial variations of the leading-order displacement (see, e.g. [7]), this assumption means that we focus on a regime where we do not expect the motion of the fluid to produce significant changes in the elastic strains of the porous medium over the timescale of the fluid flow, a condition which is typically met in the context of slowly flowing viscous fluids interacting with an almost non-deformable solid domain.

In the following section, we compare coefficients of the same power of $\epsilon ^{l}$ for $l=0,1,\dots$ to obtain conditions that allow us to determine (a) a new system of PDEs in terms of the leading (zeroth) order fields to describe the macroscale behaviour of a nematic liquid crystal in elastic porous media, (b) the development of a homogenised set of constitutive relationships that accurately depict the composite system’s overall anisotropic poro-viscoelastic behaviour. The effective coefficients preserve the distinct contributions of the solid and fluid phases, and (c) the new microscale cell problems which must be resolved in order to obtain the coefficients of the resulting homogenised model. As such, the model will retain a link between the properties of the microstructure (i.e. geometry, elastic stiffness, viscosities, and the nematic director) and the macroscale response of the material.

## Derivation of the homogenised model

In this section, we derive a closed system of PDEs for the leading-order variables $\boldsymbol{u}^{(0)}$, $\boldsymbol{v}^{(0)}$, and ${p}^{(0)}$ over the macroscale domain spanned by the variable $\boldsymbol{x}$. This is achieved by considering the multiscale system of PDEs, as well as associated constitutive relationships, (17a–17h) and (17i–17j) as polynomial expressions in $\epsilon$. As such, by identity of polynomials, we obtain a set of differential equations by equating the coefficients of $\epsilon ^l$, for $l=0,1,\ldots$.

### Remark 4

(The nematic director) Due to the symmetry of the stress tensor, the nematic director field is determined solely by the elasticity, through the balance of angular momentum, together with the director boundary conditions, and does not depend on the fluid behaviour. We present the derivation of a closed macroscale system of PDEs for the leading-order nematic director $\boldsymbol{m}^{(0)}$ in Appendix, in which the director $\boldsymbol{m}$ is considered a multiscale function, and its leading and order one component satisfy $\boldsymbol{m}^{(0)}=\boldsymbol{m}^{(0)} (\boldsymbol{x})$ and

20 $$\begin{aligned} \nabla _{ \boldsymbol{y}} \boldsymbol{m}^{(1)} = {\mathbb {G}}: \nabla _{ \boldsymbol{x}} \boldsymbol{m}^{(0)} \end{aligned}$$

respectively, where ${\mathbb {G}}$ is a fourth-rank tensor defined as

21 $$\begin{aligned} {\mathbb {G}}:= \nabla _{\boldsymbol{y}} \mathcal {W}. \end{aligned}$$

The third-rank tensor $\mathcal {W}$ depends only on the geometry of the microstructure and is to be computed by solving the cell problem (87a–88) in Appendix. These governing equations consider only the elastic free energy for determining the nematic director and use the simplifying assumption of a one elastic constant approximation, that is, we assume that the Frank elastic constants for splay director distortion, $K_1$, for twist director distortion, $K_2$, and for bend director distortions, $K_3$, are equal, so that $K_{1}=K_{2}=K_{3}=K$. However, additional effects can readily be incorporated, including the more general Oseen–Frank formulation in which these elastic constants differ, as well as the additional influence of external (e.g. magnetic or electric) fields, as shown for example, in [68]. Using this approach, the director fields $\boldsymbol{m}^{(0)}$ and $\boldsymbol{m}^{(1)}$ are subsequently treated as being completely determined.

$$\begin{aligned} \boxed {\epsilon ^0} \phantom {00000000000000000000000000000000000000000000000000000000000000000000000000000000000000} \end{aligned}$$

Equating coefficients of $\epsilon ^{0}$ in the system (17a–17j), we obtain:

22a $$\begin{aligned} \nabla _{\boldsymbol{y}} \cdot \mathsf {\sigma }_\textrm{f}^{(0)}&=0  &   \text {in } \ \Omega _{\textrm{f}}, \end{aligned}$$

22b $$\begin{aligned} \nabla _{\boldsymbol{y}} \cdot \boldsymbol{v}^{(0)}&= 0  &   \text {in }\ \Omega _{\textrm{f}}, \end{aligned}$$

22c $$\begin{aligned} \nabla _{\boldsymbol{y}} \cdot \mathsf {\sigma }^{(0)}_\textrm{s}&= 0  &   \text {in } \ \Omega _{\textrm{s}}, \end{aligned}$$

22d $$\begin{aligned} \boldsymbol{v}^{(0)}&= \boldsymbol{\dot{u}}^{(0)}  &   \text {on }\ \Gamma ,\end{aligned}$$

22e $$\begin{aligned} \mathsf {\sigma }^{(0)}_{\textrm{f}} \boldsymbol{n}&= \mathsf {\sigma }^{(0)}_{\textrm{s}} \boldsymbol{n}\quad  &   \text {on }\ \Gamma , \end{aligned}$$

22f $$\begin{aligned} - \bar{K}\left( \left( \nabla _{ \boldsymbol{y}} \boldsymbol{m}^{(0)}\right) ^{\top } \nabla _{ \boldsymbol{y}} \boldsymbol{m}^{(0)} \right)&= 0  &   \text {in }\ \Omega _{\textrm{f}}, \end{aligned}$$

22g $$\begin{aligned} {\mathbb {C}} : \textsf{D}_{ \boldsymbol{y}} ( \boldsymbol{u}^{(0)})&= 0  &   \text {in }\ \Omega _{\textrm{s}}. \end{aligned}$$

Equation (22f), as per Remark 4, is automatically satisfied (see the Appendix) since

23 $$\begin{aligned} \boldsymbol{m}^{(0)} = \boldsymbol{m}^{(0)}( \boldsymbol{x}). \end{aligned}$$

Equation (22g) implies that the symmetric part of the microscale leading-order displacement vanishes, which in turn leads to $\boldsymbol{u}^{(0)}=\boldsymbol{\omega } \wedge \textbf{y}+\bar{\boldsymbol{u}}(\boldsymbol{x})$, where $\boldsymbol{\omega }$ is a constant vector and $\bar{\boldsymbol{u}}$ is a vector-valued function that does not depend on the microscale $\boldsymbol{y}$. However, as we are requiring that $\boldsymbol{u}^{(0)}$ must be $\boldsymbol{y}$-periodic, then we simply have

24 $$\begin{aligned} \boldsymbol{u}^{(0)}=\boldsymbol{u}^{(0)}(\boldsymbol{x}), \end{aligned}$$

that is, the leading-order displacement depends on the macroscale $\boldsymbol{x}$ only.

$$\begin{aligned} \boxed {\epsilon ^1} \phantom {00000000000000000000000000000000000000000000000000000000000000000000000000000000000000} \end{aligned}$$

The differential conditions that are obtained by equating the coefficients multiplying $\epsilon ^{1}$ read:

25a $$\begin{aligned} \nabla _{ \boldsymbol{y}} \cdot \mathsf {\sigma }_{\textrm{f}}^{(1)}+\nabla _{ \boldsymbol{x}} \cdot \mathsf {\sigma }_{\textrm{f}}^{(0)}&=0 \quad  &   \text{ in } \ \Omega _{\textrm{f}}, \end{aligned}$$

25b $$\begin{aligned} \nabla _{ \boldsymbol{y}} \cdot \boldsymbol{v}^{(1)}+\nabla _{ \boldsymbol{x}} \cdot \boldsymbol{v}^{(0)}&=0 \quad  &   \text{ in } \ \Omega _{\textrm{f}}, \end{aligned}$$

25c $$\begin{aligned} \nabla _{ \boldsymbol{y}} \cdot \mathsf {\sigma }_{\textrm{s}}^{(1)}+\nabla _{ \boldsymbol{x}} \cdot \mathsf {\sigma }_{\textrm{s}}^{(0)}&=0 \quad  &   \text{ in } \ \Omega _{\textrm{s}}, \end{aligned}$$

25d $$\begin{aligned} \boldsymbol{v}^{(1)}&= 0 \quad  &   \text{ on } \ \mathrm {\Gamma },\end{aligned}$$

25e $$\begin{aligned} \mathsf {\sigma }^{(1)}_{\textrm{f}} \boldsymbol{n}&= \mathsf {\sigma }^{(1)}_{\textrm{s}} \boldsymbol{n} \quad  &   \text{ on } \ \Gamma ,\end{aligned}$$

25f $$\begin{aligned} 2 \bar{\mu } \left( \textsf{D}_{\boldsymbol{y}} (\boldsymbol{v}^{(0)}) \right) - \bar{K} \bigg ( \left( \nabla _{\boldsymbol{x}} \boldsymbol{m}^{(0)} \right) ^{\top } \nabla _{\boldsymbol{y}} \boldsymbol{m}^{(0)} + \left( \nabla _{\boldsymbol{y}} \boldsymbol{m}^{(0)} \right) ^{\top } \nabla _{\boldsymbol{x}} \boldsymbol{m}^{(0)}  &    &\nonumber \\ + \left( \nabla _{\boldsymbol{y}} \boldsymbol{m}^{(1)} \right) ^{\top } \nabla _{\boldsymbol{y}} \boldsymbol{m}^{(0)} + \left( \nabla _{\boldsymbol{y}} \boldsymbol{m}^{(0)} \right) ^{\top } \nabla _{\boldsymbol{y}} \boldsymbol{m}^{(1)} \bigg )  &    &\nonumber \\ + 2 \bar{\alpha } \bar{\mu } \left( \textsf{D}_{\boldsymbol{y}} \left( \boldsymbol{v}^{(0)} \right) \boldsymbol{m}^{(0)} \otimes \boldsymbol{m}^{(0)} \right) + 2 \bar{\alpha } \bar{\mu } \left( \boldsymbol{m}^{(0)} \otimes \textsf{D}_{\boldsymbol{y}} \left( \boldsymbol{v}^{(0)}\right) \boldsymbol{m}^{(0)} \right)  &    &\nonumber \\ +2\bar{\mu } \bar{\alpha }_{1}\bigg (\left( \boldsymbol{m}^{(0)} \cdot \textsf{D}_{\boldsymbol{y}}\left( \boldsymbol{v}^{(0)}\right) \boldsymbol{m}^{(0)}\right) \boldsymbol{m}^{(0)} \otimes \boldsymbol{m}^{(0)} \bigg )&= 0 \quad  &   \text{ in } \ \Omega _{\textrm{f}}, \end{aligned}$$

25g $$\begin{aligned} {\mathbb {C}}: \textsf{D}_{ \boldsymbol{y}} (\boldsymbol{u}^{(1)})+{\mathbb {C}} :\textsf{D}_{ \boldsymbol{x}} (\boldsymbol{u}^{(0)})&=\mathsf {\sigma }_\textrm{s}^{(0)} \quad  &   \text{ in } \ \Omega _{\textrm{s}}, \end{aligned}$$

where we have considered that non-leading-order contributions to the solid velocity, cf. Remark 3, are being neglected to obtain (25d). We notice that since $\boldsymbol{m}^{(0)}$ depends only on $\boldsymbol{x}$, then equation (25f) simplifies to

26 $$\begin{aligned} \begin{aligned} 2 \bar{\mu } \textsf{D}_{ \boldsymbol{y}}\left( \boldsymbol{v}^{(0)}\right) +2 \bar{\alpha } \bar{\mu }\left( \textsf{D}_{\boldsymbol{y}} \left( \boldsymbol{v}^{(0)}\right) \boldsymbol{m}^{(0)} \otimes \boldsymbol{m}^{(0)}\right) + 2 \bar{\alpha } \bar{\mu } \left( \boldsymbol{m}^{(0)} \otimes \textsf{D}_{\boldsymbol{y}} \left( \boldsymbol{v}^{(0)} \right) \boldsymbol{m}^{(0)}\right)&\\+ 2\bar{\mu } \bar{\alpha }_{1}\bigg (\left( \boldsymbol{m}^{(0)} \cdot \textsf{D}_{\boldsymbol{y}}\left( \boldsymbol{v}^{(0)}\right) \boldsymbol{m}^{(0)} \right) \boldsymbol{m}^{(0)} \otimes \boldsymbol{m}^{(0)} \bigg )&=0\quad \text{ in } \ \Omega _{\textrm{f}}, \end{aligned} \end{aligned}$$

or equivalently

27 $$\begin{aligned} {\mathbb {T}}( \boldsymbol{m}^{(0)}):\nabla _{ \boldsymbol{y}} \boldsymbol{v}^{(0)}= {\mathbb {T}}( \boldsymbol{m}^{(0)}): \textsf{D}_{\boldsymbol{y}}\left( \boldsymbol{v}^{(0)}\right) =0 \quad \text{ in } \ \Omega _{\textrm{f}}, \end{aligned}$$

where the minor symmetric fourth-rank operator ${\mathbb {T}}$ is defined component-wise as

28 $$\begin{aligned} \begin{aligned} T_{ijlp}( \boldsymbol{m}^{(0)})= \bar{\mu } \bigg [&\delta _{il} \delta _{jp}+\delta _{ip} \delta _{jl}+2\bar{\alpha }_{1}m^{(0)}_{i} m^{(0)}_{j}m^{(0)}_{l} m^{(0)}_{p}\\  &+\bar{\alpha } \bigg (\delta _{il} m^{(0)}_{j} m^{(0)}_{p}+\delta _{ip} m^{(0)}_{j} m^{(0)}_{l}+ m^{(0)}_{i}\delta _{jl} m^{(0)}_{p}+ m^{(0)}_{i}\delta _{jp} m^{(0)}_{l} \bigg ) \bigg ], \end{aligned} \end{aligned}$$

and the standard conditions on the Leslie viscosities which ensure positive semi-definiteness, see, e.g. Section 4.2.3 in [69], are $\bar{\mu }> 0$, $\bar{\alpha }\ge -1$ and $\bar{\alpha }_1\ge -(4\bar{\alpha }+3)/2$. For example, we could consider the form of ${\mathbb {T}}$ when fixing the director to be aligned with one of the three orthogonal axes, say, without loss of generality, by selecting $\boldsymbol{m}^{(0)}=\boldsymbol{e}_x$, so that the nonzero components of ${\mathbb {T}}$ in this case are,

29a $$\begin{aligned} T_{1111}( \boldsymbol{m}^{(0)})= 2\bar{\mu } \bigg [&1+\bar{\alpha }_{1}+2\bar{\alpha }\bigg ],&\end{aligned}$$

29b $$\begin{aligned} T_{\beta \beta \beta \beta }( \boldsymbol{m}^{(0)})= 2\bar{\mu }&,&\ \ \beta =2,3\end{aligned}$$

29c $$\begin{aligned} T_{1\beta 1\beta }( \boldsymbol{m}^{(0)})=T_{\beta 1\beta 1}( \boldsymbol{m}^{(0)})= T_{1\beta \beta 1}( \boldsymbol{m}^{(0)})=T_{\beta 11\beta }( \boldsymbol{m}^{(0)})= \bar{\mu } \bigg [&1+\bar{\alpha }\bigg ],&\ \ \beta =2,3\end{aligned}$$

29d $$\begin{aligned} T_{2323}( \boldsymbol{m}^{(0)})=T_{3232}( \boldsymbol{m}^{(0)})=T_{2332}( \boldsymbol{m}^{(0)})=T_{3223}( \boldsymbol{m}^{(0)})= \bar{\mu },&\end{aligned}$$

where in this case submission over $\beta$ is not intended. The obtained expression the symmetry of the nematic stress tensor, in that there remain two non-Newtonian viscosities, $\bar{\alpha }$, $\bar{\alpha }_1$ the first of which is involved when the director is in the shear plane and both of which are involved when the director is at an acute angle to the shear plane.

Since a general solution of equation (27) should be valid for every admissible choice of parameters and director orientation, including where $\bar{\alpha }_1=0=\bar{\alpha }$ or where ${m}^{(0)}$ is perpendicular to the shear plane, both of which are equivalent to the isotropic Newtonian limit, then $\textsf{D}_{\boldsymbol{y}}\left( \boldsymbol{v}^{(0)}\right)$ must vanish, so that, by $\boldsymbol{y}$-periodicity, we also have

30 $$\begin{aligned} \boldsymbol{v}^{(0)}=\boldsymbol{v}^{(0)} ( \boldsymbol{x}), \end{aligned}$$

i.e. the leading-order velocity $\boldsymbol{v}^{(0)}$ depends only on the macroscale.

Integrating equation (25b) over $\Omega _{\textrm{f}}$, we find that

31 $$\begin{aligned} \int _{ \Omega _\textrm{f} } \nabla _{ \boldsymbol{y}} \cdot \boldsymbol{v}^{(1)} dV= - \left| \Omega _{\textrm{f}}\right| \nabla _{ \boldsymbol{x}} \cdot \boldsymbol{v}^{(0)} \end{aligned}$$

By applying the divergence theorem with respect to the microscale $\boldsymbol{y}$ and considering that the surface integral contributions on the periodic boundaries $\partial \Omega _{\textrm{f}} \setminus \Gamma$ vanish due to $\boldsymbol{y}$-periodicity, we then obtain

32 $$\begin{aligned} \int _{ \Gamma } \boldsymbol{v}^{(1)} \cdot \boldsymbol{n}\, \textrm{dS}= \left| \Omega _{\textrm{f}}\right| \nabla _{ \boldsymbol{x}} \cdot \boldsymbol{v}^{(0)},\nonumber \\ \end{aligned}$$

where $\boldsymbol{n}$ is the unit vector normal to the interface $\Gamma$ and pointing out of the fluid domain. Since (25d) holds, we then obtain the following macroscale incompressibility constraint,

33 $$\begin{aligned} \nabla _{ \boldsymbol{x}} \cdot \boldsymbol{v}^{(0)}=0. \end{aligned}$$

Furthermore, equation (25b) yields

34 $$\begin{aligned} \nabla _{ \boldsymbol{y}} \cdot \boldsymbol{v}^{(1)}=0. \end{aligned}$$

Now we can recast the equations (25a–26) as:

35a $$\begin{aligned} \nabla _{ \boldsymbol{y}} \cdot \mathsf {\sigma }_{\textrm{f}}^{(1)}+\nabla _{ \boldsymbol{x}} \cdot \mathsf {\sigma }_{\textrm{f}}^{(0)}&=0 \quad  &   \text{ in } \ \Omega _{\textrm{f}}, \end{aligned}$$

35b $$\begin{aligned} \nabla _{ \boldsymbol{y}} \cdot \boldsymbol{v}^{(1)}&=0 \quad  &   \text{ in } \ \Omega _{\textrm{f}}, \end{aligned}$$

35c $$\begin{aligned} \nabla _{ \boldsymbol{y}} \cdot \mathsf {\sigma }_{\textrm{s}}^{(1)}+\nabla _{ \boldsymbol{x}} \cdot \mathsf {\sigma }_{\textrm{s}}^{(0)}&=0 \quad  &   \text{ in } \ \Omega _{\textrm{s}}, \end{aligned}$$

35d $$\begin{aligned} \boldsymbol{v}^{(1)}&= 0 \quad  &   \text{ on } \ \mathrm {\Gamma }, \end{aligned}$$

35e $$\begin{aligned} \mathsf {\sigma }^{(1)}_{\textrm{f}} \boldsymbol{n}&= \mathsf {\sigma }^{(1)}_{ \textrm{s}} \boldsymbol{n} \quad  &   \text{ on } \ \Gamma , \end{aligned}$$

35f $$\begin{aligned} {\mathbb {C}} : \textsf{D}_{ \boldsymbol{y}} (\boldsymbol{u}^{(1)})+{\mathbb {C}} : \textsf{D}_{ \boldsymbol{x}} (\boldsymbol{u}^{(0)})&=\mathsf {\sigma }_{\textrm{s}}^{(0)} \quad  &   \text{ in } \ \Omega _{\textrm{s}}, \end{aligned}$$

As the leading-order fluid and solid velocity are both constant with respect to the microscale $\boldsymbol{y}$, the continuity condition (22d) then also implies

36 $$\begin{aligned} \boldsymbol{v}^{(0)} ( \boldsymbol{x})=\dot{\boldsymbol{u}}^{(0)} (\boldsymbol{x}). \end{aligned}$$

$$\begin{aligned} \boxed {\epsilon ^2} \phantom {00000000000000000000000000000000000000000000000000000000000000000000000000000000000000} \end{aligned}$$

We equate the same powers of $\epsilon ^{2}$ in the system (17a- 17j) to obtain:

37a $$\begin{aligned} \nabla _{ \boldsymbol{y}} \cdot \mathsf {\sigma }_{\textrm{f}}^{(2)}+\nabla _{ \boldsymbol{x}} \cdot \mathsf {\sigma }_{\textrm{f}}^{(1)}&=0 \quad  &   \text{ in } \ \Omega _{\textrm{f}},\end{aligned}$$

37b $$\begin{aligned} \nabla _{ \boldsymbol{y}} \cdot \boldsymbol{v}^{(2)}+\nabla _{ \boldsymbol{x}} \cdot \boldsymbol{v}^{(1)}&=0 \quad  &   \text{ in } \ \Omega _{\textrm{f}},\end{aligned}$$

37c $$\begin{aligned} \nabla _{ \boldsymbol{y}} \cdot \mathsf {\sigma }_{\textrm{s}}^{(2)}+\nabla _{ \boldsymbol{x}} \cdot \mathsf {\sigma }_{\textrm{s}}^{(1)}&=0 \quad  &   \text{ in } \ \Omega _{\textrm{s}},\end{aligned}$$

37d $$\begin{aligned} \boldsymbol{v}^{(2)}&= 0 \quad  &   \text{ on } \ \mathrm {\Gamma },\end{aligned}$$

37e $$\begin{aligned} \mathsf {\sigma }^{(2)}_{\textrm{f}} \boldsymbol{n}&= \mathsf {\sigma }^{(2)}_{ \textrm{s}} \boldsymbol{n} \quad  &   \text{ on } \ \Gamma ,\end{aligned}$$

37f $$\begin{aligned} -p^{(0)} \textsf{I} - \bar{K} \Bigg ( \left( \nabla _{\boldsymbol{x}} \boldsymbol{m}^{(0)}\right) ^{\top } \nabla _{\boldsymbol{x}} \boldsymbol{m}^{(0)} + \left( \nabla _{\boldsymbol{y}} \boldsymbol{m}^{(1)}\right) ^{\top } \nabla _{\boldsymbol{y}} \boldsymbol{m}^{(1)}\nonumber  &    &\\ + \left( \nabla _{\boldsymbol{y}} \boldsymbol{m}^{(1)}\right) ^{\top } \nabla _{\boldsymbol{x}} \boldsymbol{m}^{(0)} + \left( \nabla _{\boldsymbol{x}} \boldsymbol{m}^{(0)}\right) ^{\top } \nabla _{\boldsymbol{y}} \boldsymbol{m}^{(1)} \Bigg ) \nonumber  &    &\\ + {\mathbb {T}}( \boldsymbol{m}^{(0)}):\nabla _{ \boldsymbol{x}} \boldsymbol{v}^{(0)}+{\mathbb {T}}( \boldsymbol{m}^{(0)}):\nabla _{ \boldsymbol{y}} \boldsymbol{v}^{(1)}&=\mathsf {\sigma }_{\textrm{f}}^{(0)} \quad  &   \text { in } \ \Omega _{\textrm{f}}\end{aligned}$$

37g $$\begin{aligned} {\mathbb {C}}: \textsf{D}_{ \boldsymbol{y}} (\boldsymbol{u}^{(2)})+{\mathbb {C}} : \textsf{D}_{ \boldsymbol{x}} (\boldsymbol{u}^{(1)})&=\mathsf {\sigma }_{\textrm{s}}^{(1)}\quad  &   \text{ in } \ \Omega _{\textrm{s}}, \end{aligned}$$

where we have neglected terms involving differentiation with respect to the microscale variable $\boldsymbol{y}$ with respect to $\boldsymbol{m}^{(0)}$ and $\boldsymbol{v}^{(0)}$ as these depend only on $\boldsymbol{x}$.

Following the Appendix, the leading-order fluid stress tensor can also be rewritten by accounting for the relationship between the first-order and leading-order nematic director, as per Remark 4. By substituting equation (20) into (37f) yields

38a $$\begin{aligned} \mathsf {\sigma }_{\textrm{f}}^{(0)}&=-p^{(0)}\, \textsf{I} - \texttt{S}\boldsymbol{:}{\mathbb {Q}} + {\mathbb {T}}( \boldsymbol{m}^{(0)}):\nabla _{ \boldsymbol{x}} \boldsymbol{v}^{(0)}+{\mathbb {T}}( \boldsymbol{m}^{(0)}):\nabla _{ \boldsymbol{y}} \boldsymbol{v}^{(1)} \quad  &   \text {in } \ \Omega _{\textrm{f}}, \end{aligned}$$

38b $$\begin{aligned} \texttt{S}\boldsymbol{:}{\mathbb {Q}}&=\bar{K}\bigg (\left( \nabla _{ \boldsymbol{x}} \boldsymbol{m}^{(0)}\right) ^{\top } \nabla _{ \boldsymbol{x}} \boldsymbol{m}^{(0)} {\mathbb {G}}+{\mathbb {G}}^{\top } \left( \nabla _{ \boldsymbol{x}} \boldsymbol{m}^{(0)}\right) ^{\top } \nabla _{ \boldsymbol{x}} \boldsymbol{m}^{(0)}&\nonumber \\&\quad \quad +{\mathbb {G}}^{\top } \left( \nabla _{ \boldsymbol{x}} \boldsymbol{m}^{(0)}\right) ^{\top } \nabla _{ \boldsymbol{x}} \boldsymbol{m}^{(0)} {\mathbb {G}}+\left( \nabla _{\boldsymbol{x}} \boldsymbol{m}^{(0)}\right) ^{\top } \nabla _{\boldsymbol{x}} \boldsymbol{m}^{(0)} \bigg ) \quad  &   \text {in } \ \Omega _{\textrm{f}}, \end{aligned}$$

where the fourth-rank tensor ${\mathbb {Q}}$ is defined as:

39 $$\begin{aligned} {\mathbb {Q}}=\nabla _{ \boldsymbol{x}} \boldsymbol{m}^{(0)} \otimes \nabla _{ \boldsymbol{x}} \boldsymbol{m}^{(0)}. \end{aligned}$$

and the sixth-rank tensor $\texttt{S}$ is defined component-wise as

40 $$\begin{aligned} S_{ijlmpq}= \bar{K}\left( \delta _{pl} \delta _{jq} \delta _{im}+\delta _{kp} \delta _{jq} G_{kilm}+\delta _{kl} \delta _{im} G_{kjpq}+G_{kilm} G _{kjpq}\right) . \end{aligned}$$

The operation “$\boldsymbol{:}$” that is shown in (38b) is a quadrupole contraction between tensors of rank four or higher, for instance the left-hand side of relationship (38b) reads, component-wise: $S_{ijlmpq}Q_{lmpq}$.

### Balance of linear momentum at the macroscale

In order to obtain the balance of linear momentum at the macroscale, we start by summing up the integral averages of equations (35a) and (35c) on the corresponding cell portions $\Omega _\textrm{f}$ and $\Omega _\textrm{s}$ such that

41 $$\begin{aligned} \int _{\Omega _{\textrm{f}}} \nabla _{\boldsymbol{y}} \cdot \mathsf {\sigma }_{\textrm{f}}^{(1)} \textrm{d} \boldsymbol{y}+\int _{\Omega _{\textrm{s}}} \nabla _{\boldsymbol{y}} \cdot \mathsf {\sigma }_{\textrm{s}}^{(1)} \textrm{d} \boldsymbol{y}+\int _{\Omega _{\textrm{f}}} \nabla _{ \boldsymbol{x}} \cdot \mathsf {\sigma }_{\textrm{f}}^{(0)} \textrm{d} \boldsymbol{y}+\int _{\Omega _{\textrm{s}}} \nabla _{ \boldsymbol{x}} \cdot \mathsf {\sigma }_{\textrm{s}}^{(0)} \textrm{d} \boldsymbol{y}=0. \end{aligned}$$

By applying the divergence theorem with respect to $\boldsymbol{y}$ to the first and second integral, and by accounting for macroscopic uniformity as per Remark 2 in the last two integrals we obtain

42 $$\begin{aligned} \begin{aligned} \int _{\partial \Omega _{\textrm{f}} / \Gamma } \mathsf {\sigma }_{\textrm{f}}^{(1)} \boldsymbol{n}_{\Omega _{\textrm{f}}} \textrm{dS}+\int _{\Gamma } \mathsf {\sigma }_{\textrm{f}}^{(1)} \boldsymbol{n} \textrm{dS}+\int _{\partial \Omega _{\textrm{s}} / \Gamma } \mathsf {\sigma }_{\textrm{s}}^{(1)} \boldsymbol{n}_{\Omega _{\textrm{s}}} \textrm{dS}+\int _{\Gamma } \mathsf {\sigma }_{\textrm{s}}^{(1)} \boldsymbol{n}_{\textrm{s}} \textrm{dS}+\nabla _{ \boldsymbol{x}} \cdot \int _{\Omega _{\textrm{f}}} \mathsf {\sigma }_{\textrm{f}}^{(0)} d \boldsymbol{y}+\nabla _{ \boldsymbol{x}} \cdot \int _{\Omega _{\textrm{s}}} \mathsf {\sigma }_{\textrm{s}}^{(0)} d \boldsymbol{y}=0, \end{aligned} \end{aligned}$$

where $\boldsymbol{n}_{\Omega _{\textrm{f}}}$ and $\boldsymbol{n}_{\Omega _{\textrm{s}}}$ are the unit normal vectors to the periodic cell boundaries $\partial \Omega _{\textrm{f}} \setminus \Gamma$ and $\partial \Omega _{\textrm{s}} \setminus \Gamma$, respectively. Since the contributions over $\partial \Omega _{\textrm{f}} \setminus \Gamma$ and $\partial \Omega _{\textrm{s}} \setminus \Gamma$ cancel because of $\boldsymbol{y}$-periodicity, we can recast equation (42) as follows

43 $$\begin{aligned} \begin{aligned} \int _{\Gamma } \mathsf {\sigma }_{\textrm{f}}^{(1)} \boldsymbol{n} \textrm{dS}+\int _{\Gamma } \mathsf {\sigma }_{\textrm{s}}^{(1)} \boldsymbol{n}_{\textrm{s}} \textrm{dS}+\nabla _{ \boldsymbol{x}} \cdot \int _{\Omega _{\textrm{f}}} \mathsf {\sigma }_{\textrm{f}}^{(0)} d \boldsymbol{y}+\nabla _{ \boldsymbol{x}} \cdot \int _{\Omega _{\textrm{s}}} \mathsf {\sigma }_{\textrm{s}}^{(0)} d \boldsymbol{y}=0, \end{aligned} \end{aligned}$$

where $\boldsymbol{n}_\textrm{s}= -\boldsymbol{n}$ and therefore from equation (35e), the first and second integrals in (43) vanish and we are left with

44 $$\begin{aligned} \nabla _{ \boldsymbol{x}} \cdot \left\langle \sigma _{\textrm{f}}^{(0)}\right\rangle _{\textrm{f}}+\nabla _{ \boldsymbol{x}} \cdot \left\langle \sigma _{\textrm{s}}^{(0)}\right\rangle _{\textrm{s}}={0}. \end{aligned}$$

We now need to express the macroscale balance of linear momentum, and therefore, both the fluid and the solid leading-order average stress tensors, in terms of the leading-order fields $\boldsymbol{u}^{(0)}, \boldsymbol{v}^{(0)}, p^{(0)}, \boldsymbol{m}^{(0)}$.

### The leading-order fluid stress tensor

We now proceed in finding an expression for the first-order velocity $\boldsymbol{v}^{(1)}$ in terms of leading-order fields only. By substituting the leading-order fluid stress tensor (38a) into the microscale stress balance equation (22a) and by taking into account that $\boldsymbol{m}^{(0)}$ and $\boldsymbol{v}^{( 0)}$ are independent of $\boldsymbol{y}$, we now have the stress balance, together with the incompressibility constraint (35b) and interface condition (35d), rewritten in terms of $\boldsymbol{v}^{(1)}$ and $p^{(0)}$:

45a $$\begin{aligned} \nabla _{\boldsymbol{y}} \cdot \Big ({\mathbb {T}}( \boldsymbol{m}^{(0)}):\nabla _{\boldsymbol{y}}\boldsymbol{v}^{(1)}\Big )- \nabla _{\boldsymbol{y}} p^{(0)} - \nabla _{\boldsymbol{y}} \cdot \left( \texttt{S}: {\mathbb {Q}} \right)&=0 \quad  &   \text { in } \ \Omega _{\textrm{f}},\end{aligned}$$

45b $$\begin{aligned} \nabla _{ \boldsymbol{y}} \cdot \boldsymbol{v}^{(1)}&=0 \quad  &   \text{ in } \ \Omega _{\textrm{f}}, \end{aligned}$$

45c $$\begin{aligned} \boldsymbol{v}^{(1)}&= 0 \quad  &   \text{ on } \ \mathrm {\Gamma }. \end{aligned}$$

Since $\nabla _{\boldsymbol{x}} \boldsymbol{m}^{(0)}$, and hence ${\mathbb {Q}}$, depend only on $\boldsymbol{x}$, by linearity we can state the following ansätze for the solution of the system (45a–45c),

46a $$\begin{aligned} \boldsymbol{v}^{(1)}&=\boldsymbol{\textsf{A}}\boldsymbol{:} {\mathbb {Q}},\end{aligned}$$

46b $$\begin{aligned} p^{(0)}&={\mathbb {B}}\boldsymbol{:}{\mathbb {Q}}+\tilde{p}(\boldsymbol{x}), \end{aligned}$$

where the auxiliary quantities $\boldsymbol{\textsf{A}}$ and ${\mathbb {B}}$ are tensors of fifth and fourth rank, respectively, and $\tilde{p}(\boldsymbol{x})$ is a $\boldsymbol{y}$-independent scalar field. The fields $\boldsymbol{\textsf{A}}$ and ${\mathbb {B}}$ satisfy the following periodic cell problems

47a $$\begin{aligned} \nabla _{ \boldsymbol{y}}\cdot \left( {\mathbb {T}}( \boldsymbol{m}^{(0)}):\nabla _{\boldsymbol{y}} \boldsymbol{\textsf{A}}\right) -\nabla _{\boldsymbol{y}} {\mathbb {B}}-\nabla _{\boldsymbol{y}} \cdot \texttt{S}&=0 \quad  &   \text{ in } \ \Omega _{\textrm{f}},\end{aligned}$$

47b $$\begin{aligned} \nabla _{\boldsymbol{y}}\cdot \boldsymbol{\textsf{A}}&=0 \quad  &   \text{ in } \ \Omega _{\textrm{f}},\end{aligned}$$

47c $$\begin{aligned} \boldsymbol{\textsf{A}}&=0 \quad  &   \text{ on } \ \Gamma , \end{aligned}$$

equipped with periodic conditions on $\partial \Omega _\textrm{f} \setminus \Gamma$. Non-trivial solutions of the problem are driven by the microscale divergence of the sixth-order tensor $\texttt{S}$, which depends on the microscale geometry through the fourth-rank tensor ${\mathbb {G}}$ that is specified in Appendix, as well as the elastic nematic energy, cf. definition (40). We also require one further condition on the auxiliary variable ${\mathbb {B}}$, which is only determined up to an additive constant, and so set

48 $$\begin{aligned} \langle {\mathbb {B}} \rangle _{{\textrm{f}}}=0. \end{aligned}$$

The above differential problem can be written component-wise as follows:

49a $$\begin{aligned} \frac{\partial }{\partial {y}_j}\left( T_{ijrs} \frac{\partial A_{rlmpq}}{\partial y_{s}}\right) -\frac{\partial B_{lmpq}}{\partial {y}_i}- \frac{\partial S_{ijlmpq}}{\partial {y}_j}&=0 \quad  &   \text{ in } \ \Omega _{\textrm{f}},\end{aligned}$$

49b $$\begin{aligned} \frac{\partial }{\partial {y}_i}\left( A_{ilmpq}\right)&=0 \quad  &   \text{ in } \ \Omega _{\textrm{f}},\end{aligned}$$

49c $$\begin{aligned} A_{ilmpq}&=0 \quad  &   \text{ on } \ \Gamma . \end{aligned}$$

As such, the system of PDEs for the auxiliary fifth- and fourth-rank tensors $(\textsf{A}, {\mathbb {B}})$ corresponds to one anisotropic fluid flow periodic cell problem (encoding the role of the Leslie viscosities $\mu$, $\alpha _1$, and $\alpha$) for each fixed set of indices (*lmpq*), where $l,m,p,q=1,..n$, so that in principle $n^4$ for $n=2$ or 3 are to be solved. Each problem is equipped with a corresponding incompressibility constraint and no-slip condition on the interface $\Gamma$. By substituting the ansätze (46a–46b) into the leading-order fluid stress tensor constitutive relationship (38a) and by applying the cell average operator (18), we obtain

50 $$\begin{aligned} \left\langle \mathsf {\sigma }^{(0)}_{\textrm{f}}\right\rangle _{\textrm{f}}=\left\langle ({\mathbb {T}}( \boldsymbol{m}^{(0)}):\nabla _{ \boldsymbol{y}} \boldsymbol{\textsf{A}})- \textsf{I} \otimes {\mathbb {B}}- \texttt{S}\right\rangle _{\textrm{f}}\boldsymbol{:}{\mathbb {Q}} -\phi _\textrm{f}\tilde{p} \textsf{I}+\phi _\textrm{f}\left( {\mathbb {T}}( \boldsymbol{m}^{(0)}):\textsf{D}_{\boldsymbol{x}} (\boldsymbol{v}^{(0)})\right) , \end{aligned}$$

or component-wise

51 $$\begin{aligned} \Big (\left\langle \mathsf {\sigma }^{(0)}_{\textrm{f}}\right\rangle _{\textrm{f}}\Big )_{ij}=\left\langle T_{ijrs}\frac{\partial A_{rlmpq}}{\partial y_{s}}-\delta _{ij}B_{lmpq}-S_{ijlmpq} \right\rangle _{\textrm{f}}Q_{lmpq} -\phi _\textrm{f}\tilde{p} \delta _{ij}+\phi _\textrm{f}\left( T_{ijrs} \frac{\partial v^{(0)}_{r}}{\partial y_{s}} \right) , \end{aligned}$$

where $\phi _\textrm{f}$ is the fluid volume fraction, $\phi _\textrm{f}=\left| \Omega _\textrm{f}\right| /\left| \Omega \right|$, and ${\mathbb {T}}( \boldsymbol{m}^{(0)}):\nabla _{\boldsymbol{x}} \boldsymbol{v}^{(0)}={\mathbb {T}}( \boldsymbol{m}^{(0)}):\textsf{D}_{\boldsymbol{x}} (\boldsymbol{v}^{(0)})$ as ${\mathbb {T}}$ is minor symmetric. The average leading-order stress tensor (50) is now expressed in terms of leading-order fields only, namely the zeroth-order velocity $\boldsymbol{v}^{(0)}$, nematic director $\boldsymbol{m}^{(0)}$, and the macroscale component of the pressure $\tilde{p}$. In the next section, we focus on the derivation of a corresponding expression for the solid phase stress tensor.

### The leading-order solid stress tensor

In this section, we aim to obtain a representation of the leading-order solid stress tensor in terms of leading-order fields only. By considering the leading-order fluid stress tensor relationship (38a) in terms of the ansätze (46a–46b), then equations (22c), (22e), and (35f) can be combined to write a closed system of PDEs for the order one displacement $\boldsymbol{u}^{(1)}$ as follows:

52a $$\begin{aligned} \nabla _{\boldsymbol{y}} \cdot \left( {\mathbb {C}} \textsf{D}_{\boldsymbol{y}}(\boldsymbol{u}^{(1)})\right) +\nabla _{\boldsymbol{y}} \cdot \left( {\mathbb {C}} \textsf{D}_{\boldsymbol{x}}(\boldsymbol{u}^{(0)})\right)&=0 \quad  &   \text{ in } \ \Omega _{\textrm{s}}, \end{aligned}$$

52b $$\begin{aligned} \bigg ({\mathbb {C}} \textsf{D}_{\boldsymbol{y}} (\boldsymbol{u}^{(1)})+ {\mathbb {C}} \textsf{D}_{\boldsymbol{x}} (\boldsymbol{u}^{(0)}) \bigg ) \boldsymbol{n}&= \bigg ( -\tilde{p}(\boldsymbol{x})\textsf{I}+\left( ({\mathbb {T}}( \boldsymbol{m}^{(0)}):\nabla _{ \boldsymbol{y}} \boldsymbol{\textsf{A}})- \textsf{I} \otimes {\mathbb {B}}- \texttt{S}\right) \boldsymbol{:}{\mathbb {Q}}\nonumber \\  &\quad \quad +{\mathbb {T}}(\boldsymbol{m}^{(0)}):\textsf{D}_{\boldsymbol{x}} (\boldsymbol{v}^{(0)})\bigg )\boldsymbol{n} \quad  &   \text{ on } \ \Gamma . \end{aligned}$$

Due to the linearity of the above system of PDEs (52a–52b), and since $\boldsymbol{v}^{(0)}$, $\boldsymbol{u}^{(0)}$, ${\mathbb {Q}}$, and $\tilde{p}$ do not depend on the microscale variable $\boldsymbol{y}$, we can formulate the following ansatz for $\boldsymbol{u}^{(1)}$,

53 $$\begin{aligned} \boldsymbol{u}^{(1)}= \mathcal {Z}: \textsf{D}_{\boldsymbol{x}} (\boldsymbol{u}^{(0)})+ \boldsymbol{\textsf{H}}:{\mathbb {Q}}+ \mathcal {F}:\textsf{D}_{\boldsymbol{x}} (\boldsymbol{v}^{(0)})+\boldsymbol{a}\tilde{p}(\boldsymbol{x})+\boldsymbol{c}(\boldsymbol{x}), \end{aligned}$$

where we note that the solution is only unique up to a $\boldsymbol{y}$-independent vector-valued function, which is herein denoted by $\boldsymbol{c}(\boldsymbol{x})$. The solution (53) is expressed in terms of the auxiliarry fields $\mathcal {Z}$ and $\mathcal {F}$, which are third-rank tensors, the vector $\boldsymbol{a}$, and the fifth-rank tensor $\boldsymbol{\textsf{H}}$. The following subsections address the different types of periodic cell problems which are solved by these auxiliary fields.

We start by presenting elastic-type cell problems that are independent of the nematic director, highlighting their analogy with classical poroelasticity. This is followed by a discussion concerning those elastic-type cell problems whose solution is driven by the nematic director and elastic energy. We then conclude this section by presenting the derivation of the leading-order solid stress tensor.

#### Elastic cell problems independent of the director - analogy with classical poroelasticity

The auxiliary field $\mathcal {Z}$, introduced in (53), solves the following periodic cell problem,

54a $$\begin{aligned} \nabla _{\boldsymbol{y}} \cdot \left( {\mathbb {C}} \textsf{D}_{\boldsymbol{y}}\left( \mathcal {Z}\right) \right) +\nabla _{\boldsymbol{y}} \cdot {\mathbb {C}}&=0 \quad  &   \text{ in } \ \Omega _{\textrm{s}},\end{aligned}$$

54b $$\begin{aligned} \left( {\mathbb {C}} \textsf{D}_{\boldsymbol{y}}( \mathcal {Z})+ {\mathbb {C}} \right) \boldsymbol{n}&= 0 \quad  &   \text{ on } \ \Gamma , \end{aligned}$$

while the system of PDEs for the vector field $\boldsymbol{a}$, introduced in (53), is given by,

55a $$\begin{aligned} \nabla _{\boldsymbol{y}} \cdot \left( {\mathbb {C}} \textsf{D}_{\boldsymbol{y}}\left( \boldsymbol{a}\right) \right)&=0 \quad  &   \text{ in } \ \Omega _{\textrm{s}},\end{aligned}$$

55b $$\begin{aligned} \left( {\mathbb {C}} \textsf{D}_{\boldsymbol{y}} (\boldsymbol{a})+ \textsf{I} \right) \boldsymbol{n}&= 0 \quad  &   \text{ on } \ \Gamma . \end{aligned}$$

We also require further conditions on the auxiliary variables $\mathcal {Z}$, and $\boldsymbol{a}$, which are only determined up to additive constants, and so set

56 $$\begin{aligned} \langle \mathcal {Z}\rangle _{\textrm{s}}=0, \quad \langle \boldsymbol{a}\rangle _{\textrm{s}}=0. \end{aligned}$$

The above problems can be rewritten in component form as follows

57a $$\begin{aligned} \frac{\partial }{\partial y_{j}} \left( C_{ijpq} D_{pq}^{kl}\left( Z\right) \right) +\frac{\partial C_{ijkl}}{\partial y_{j}}&=0 \quad  &   \text{ in } \ \Omega _{\textrm{s}}, \end{aligned}$$

57b $$\begin{aligned} \left( C_{ijpq} D_{pq}^{kl}\left( Z\right) + C_{ijkl} \right) n_{j}&= 0 \quad  &   \text{ on } \ \Gamma , \end{aligned}$$

57c $$\begin{aligned} \frac{\partial }{\partial y_{j}} \bigg (C_{ijpq} D_{pq}\left( a \right) \bigg )&=0 \quad  &   \text{ in } \ \Omega _{\textrm{s}}, \end{aligned}$$

57d $$\begin{aligned} \bigg (C_{ijpq} D_{pq}\left( a\right) +\delta _{ij}\bigg )n_{j}&= 0 \quad  &   \text{ on } \ \Gamma , \end{aligned}$$

where

58 $$\begin{aligned} D_{pq}(a)=(\textsf{D}_{\boldsymbol{y}} (\boldsymbol{a}))_{pq}=\frac{1}{2}\left( \frac{\partial a_p}{\partial y_q}+\frac{\partial a_q}{\partial y_p} \right) ,\, D_{pq}^{kl}(Z)=(\textsf{D}_{\boldsymbol{y}} \mathcal {Z})_{pqkl}=\frac{1}{2}\left( \frac{\partial Z_{pkl}}{\partial y_q}+\frac{\partial Z_{qkl}}{\partial y_p} \right) . \end{aligned}$$

The cell problem (54a–54b) reads as the classical periodic cell problem that arises when upscaling the standard fluid–structure interaction problem in the context of classical poroelasticity for Newtonian fluids, see, e.g. [6, 14, 44, 52, 55]. The problem, which is driven by the geometry and the original stiffness of the elastic domain, corresponds to six (or three when considering a two dimensional case) elastic-type cell problem (one for each couple of fixed indexes (*k*, *l*) by considering minor symmetry of the elasticity tensor ${\mathbb {C}}$), which are to be solved on the solid portion of the periodic cell. Examples of numerical solutions of such problems can be found, for instance, in [25], as well as their extensions to more complex scenarios involving multiple solid and fluid constituents, as in [45] and [42]. The problem (55a–55b) is an elastic-type cell problem, which is likewise analogous to the one arising when deriving Biot poroelasticity from microstructure and, in that context, it plays a role in influencing the macroscale fluid pressure, as well as other poroelastic parameters such as the Biot modulus and Biot coefficients.

In the next section, we discuss the cell problems which are affected by the liquid crystal nematic director and elastic energy.

#### Elastic-type cell problems driven by the nematic director and elastic energy

The auxiliary field $\boldsymbol{\textsf{H}}$, introduced in (53), solves the following cell problem

59a $$\begin{aligned} \nabla _{\boldsymbol{y}} \cdot \left( {\mathbb {C}} \textsf{D}_{\boldsymbol{y}}\left( \boldsymbol{\textsf{H}}\right) \right)&=0\quad  &   \text{ in } \ \Omega _{\textrm{s}},\end{aligned}$$

59b $$\begin{aligned} \left( {\mathbb {C}} \textsf{D}_{\boldsymbol{y}} (\boldsymbol{\textsf{H}})- ({\mathbb {T}}( \boldsymbol{m}^{(0)}):\nabla _{ \boldsymbol{y}} \boldsymbol{\textsf{A}})+ \textsf{I} \otimes {\mathbb {B}}+\texttt{S}\right) \boldsymbol{n}&= 0\quad  &   \text{ on } \ \Gamma , \end{aligned}$$

while the system of PDEs for the third-rank tensor field $\mathcal {F}$ in (53) reads

60a $$\begin{aligned} \nabla _{\boldsymbol{y}} \cdot \left( {\mathbb {C}} \textsf{D}_{\boldsymbol{y}}\left( \mathcal {F}\right) \right)&=0 \quad  &   \text{ in } \ \Omega _{\textrm{s}}, \end{aligned}$$

60b $$\begin{aligned} \left( {\mathbb {C}} \textsf{D}_{\boldsymbol{y}} (\mathcal {F})-{\mathbb {T}}(\boldsymbol{m}^{(0)}) \right) \boldsymbol{n}&= 0 \quad  &   \text{ on } \ \Gamma . \end{aligned}$$

Once again we require one further condition on the auxiliary variables, and we impose

61 $$\begin{aligned} \langle \mathbf {\textsf{H}}\rangle _{\textrm{s}}=0, \quad \langle \mathcal {F}\rangle _{\textrm{s}}=0. \end{aligned}$$

The component-wise representation of the problem reads

62a $$\begin{aligned} \frac{\partial }{\partial y_{j}} \bigg (C_{ijrs} D_{rs}^{lmpq}\left( H\right) \bigg )&=0 \quad  &   \text{ in } \ \Omega _{\textrm{s}},\end{aligned}$$

62b $$\begin{aligned} \left( C_{ijrs} D_{rs}^{lmpq}\left( H\right) + \delta _{ij}B_{lmpq} + S_{ijlmpq}-T(m^{(0)})_{ijrs}\frac{\partial A_{rlmpq}}{\partial y_{s}} \right) n_{j}&= 0 \quad  &   \text{ on } \ \Gamma ,\end{aligned}$$

62c $$\begin{aligned} \frac{\partial }{\partial y_{j}} \left( C_{ijpq} D_{pq}^{kl}\left( F\right) \right)&=0 \quad  &   \text{ in } \ \Omega _{\textrm{s}},\end{aligned}$$

62d $$\begin{aligned} \left( C_{ijpq} D_{pq}^{kl}\left( F\right) -T_{ijkl}(m^{(0)}) \right) n_{j}&= 0 \quad  &   \text{ on } \ \Gamma , \end{aligned}$$

where

63 $$\begin{aligned} D^{lmpq}_{rs}(H)=(\textsf{D}_{\boldsymbol{y}}\left( \boldsymbol{\textsf{H}}\right) )_{rslmpq}=\frac{1}{2}\left( \frac{\partial H_{rlmpq}}{\partial y_s}+\frac{\partial H_{slmpq}}{\partial y_r} \right) ,\, D_{pq}^{kl}(F)=(\textsf{D}_{\boldsymbol{y}} \mathcal {F})_{pqkl}=\frac{1}{2}\left( \frac{\partial F_{pkl}}{\partial y_q}+\frac{\partial F_{qkl}}{\partial y_p} \right) .\nonumber \\ \end{aligned}$$

The problem (59a–59b) reads as an elastic-type periodic cell problem, equipped with inhomogeneous Neumann interface conditions, for each fixed set of indices (*lmpq*), where $l,m,p,q=1,..n$, so that once again $n^4$ problems, for $n=2$ or 3, are to be solved. Non-trivial solutions of the problem are driven by the interface conditions, which depends on (a) the geometry of the periodic cell, (b) the constitutive relationship of the fluid and therefore the leading-order nematic director through ${\mathbb {T}}(\boldsymbol{m}^{(0)})$, and (c) on the nematic elastic energy, both directly, through the tensor $\texttt{S}$, cf. definition (40), and through the solution $(\boldsymbol{\textsf{A}}, {\mathbb {B}})$ of the problem (47a-47c). In particular, it is clear that this problem for $(\boldsymbol{\textsf{A}}, {\mathbb {B}})$ is driven by the nematic elastic energy since whenever $\bar{K}=0$, then $\texttt{S}=0$, which leads to a trivial solution of the problem (47a–47c) and in turn (59a–59b). The problem also depends on the structure of the governing equations for the nematic director through the auxiliary tensor ${\mathbb {G}}$ (cf. (40)), which is to be computed by solving the cell problems discussed in Appendix. The problem (60a–60b) corresponds to $n^2$, for $n=2$ or 3, elastic-type cell problems which are to be solved by considering interface conditions which depend on both the geometry and the fluid constitutive equation governing the fluid. In this case, the leading-order nematic director plays a role, but here there are non-trivial solutions even for a Newtonian fluid, and this problem does not encode any contribution related to the nematic elastic energy.

In the next section, we state the functional form of the leading-order stress tensor in terms of leading-order fields only.

#### The functional form of the leading-order solid stress tensor

By substituting the ansatz (53) into the relationship for the leading-order stress tensor (35f), we may now obtain the average leading-order solid stress tensor in terms of leading-order fields only, that is

64 $$\begin{aligned} \begin{aligned} \mathsf {\sigma }_{\textrm{s}}^{(0)}= ({\mathbb {C}}: {\mathbb {L}}): \textsf{D}_{\boldsymbol{x}} (\boldsymbol{u}^{(0)})+ {\mathbb {C}}:\textsf{D}_{\boldsymbol{x}}(\boldsymbol{u}^{(0)})+({\mathbb {C}}:\mathtt {\Pi })\boldsymbol{:}{\mathbb {Q}}+ ({\mathbb {C}}:\textsf{O})\tilde{p}+ ({\mathbb {C}}:{\mathbb {N}}):\textsf{D}_{ \boldsymbol{x}} (\boldsymbol{v}^{(0)}), \end{aligned} \end{aligned}$$

where ${\mathbb {L}}$ and ${\mathbb {N}}$ are fourth-rank tensors, $\mathtt {\Pi }$ is a sixth-rank tensor, and $\textsf{O}$ is second-rank tensor. Their definitions in terms of the microscale gradients of the auxiliary quantities, which appeared in the cell problems discussed above, are

65 $$\begin{aligned} {\mathbb {L}}=\textsf{D}_{\boldsymbol{y}} (\mathcal {Z}), \quad {\mathbb {N}}=\textsf{D}_{\boldsymbol{y}} (\mathcal {F}), \quad \mathtt {\Pi }=\textsf{D}_{\boldsymbol{y}} (\boldsymbol{\textsf{H}}), \quad \textsf{O}=\textsf{D}_{\boldsymbol{y}} (\boldsymbol{a}). \end{aligned}$$

Subsequently, by applying the integral average operator (18) to equation (64) over the solid domain, we obtain the leading-order average solid stress tensor,

66 $$\begin{aligned} \begin{aligned} \left\langle \mathsf { \sigma }_{\textrm{s}}^{(0)} \right\rangle _{{\textrm{s}}}= \left\langle {\mathbb {C}}: {\mathbb {L}}+{\mathbb {C}} \right\rangle _{{\textrm{s}}}:\textsf{D}_{\boldsymbol{x}} (\boldsymbol{u}^{(0)})+ \left\langle {\mathbb {C}}:\mathtt {\Pi }\right\rangle _{{\textrm{s}}}\boldsymbol{:}{\mathbb {Q}}+ \left\langle {\mathbb {C}}:\textsf{O} \right\rangle _{{\textrm{s}}} \tilde{p} +\left\langle {\mathbb {C}}:{\mathbb {N}}\right\rangle _{{\textrm{s}}}:\textsf{D}_{\boldsymbol{x}} ( \boldsymbol{v}^{(0)}), \end{aligned} \end{aligned}$$

or, component-wise,

67 $$\begin{aligned} \begin{aligned} \left( \,\left\langle \sigma _{\textrm{s}}^{(0)} \right\rangle _{{\textrm{s}}}\,\right) _{ij}= \left\langle C_{ijrs} L_{rslm}+C_{ijlm} \right\rangle _{{\textrm{s}}} D_{x_{m}} (u_{l}^{(0)}) +\left\langle C_{ijrs}\Pi _{rslmpq}\right\rangle _{{\textrm{s}}} Q_{lmpq}&\\+\left\langle C_{ijlm}O_{lm} \right\rangle _{{\textrm{s}}} \tilde{p} +\left\langle C_{ijrs} N_{rslm} \right\rangle _{{\textrm{s}}} {D}_{ x_{m}}(v_{l}^{(0)}). \end{aligned} \end{aligned}$$

We are now able to use the expressions we have derived for both the fluid and solid stress tensors to write the homogenised macroscale balance of linear momentum (44).

## Main results and discussion

We now substitute the average solid and fluid stresses, as per equations (50) and (66), into the macroscale linear momentum balance (44) to obtain the system of PDEs describing the behaviour of the nematic liquid crystal through an elastic porous medium for $\boldsymbol{x} \in \Omega _{M}$, where $\Omega _{M}$ represents the domain spanned by the macroscale variable $\boldsymbol{x}$,

68a $$\begin{aligned} \nabla _{ \boldsymbol{x}} \cdot \mathsf {\sigma }_{\text {Eff}}+\tilde{\boldsymbol{h}}&=0\quad  &   \text { in } \ \Omega _{\textrm{f}},\end{aligned}$$

68b $$\begin{aligned} \nabla _{ \boldsymbol{x}} \cdot \boldsymbol{v}^{(0)}&=0\quad  &   \text { in } \ \Omega _{\textrm{f}}, \end{aligned}$$

where the effective stress tensor is defined as

69 $$\begin{aligned} \mathsf {\sigma }_{\text {Eff}}= \left( \phi _\textrm{f} {\mathbb {T}}( \boldsymbol{m}^{(0)}) +\left\langle {\mathbb {C}}:{\mathbb {N}} \right\rangle _{\textrm{s}}\right) :\textsf{D}_{ \boldsymbol{x}}(\boldsymbol{v} ^{(0)}) + \left( \left\langle {\mathbb {C}}:\textsf{O} \right\rangle _{{\textrm{s}}}-\phi _\textrm{f} \textsf{I}\right) \tilde{p} + \left\langle {\mathbb {C}}: {\mathbb {L}}+{\mathbb {C}} \right\rangle _{{\textrm{s}}}:\textsf{D}_{\boldsymbol{x}} (\boldsymbol{u}^{(0)}), \end{aligned}$$

and the volume load is

70 $$\begin{aligned} \tilde{\boldsymbol{h}}=\tilde{\boldsymbol{g}}+ \nabla _{\boldsymbol{x}} \cdot \bigg (\left\langle {\mathbb {C}}: \mathtt {\Pi }\right\rangle _{{\textrm{s}}}\boldsymbol{:}{\mathbb {Q}}\left( \nabla _{ \boldsymbol{x}} \boldsymbol{m}\right) \bigg ), \end{aligned}$$

with

71 $$\begin{aligned} \tilde{\boldsymbol{g}}=\nabla _{\boldsymbol{x}} \cdot \Big (\left\langle ({\mathbb {T}}( \boldsymbol{m}^{(0)}):\nabla _{ \boldsymbol{y}} \boldsymbol{\textsf{A}})- \textsf{I} \otimes {\mathbb {B}}- \texttt{S}\right\rangle _{\textrm{f}}\boldsymbol{:}{\mathbb {Q}}\Big ). \end{aligned}$$

This newly derived macroscale governing system of partial differential equations reads as *anisotropic poro-viscoelastic model*, in terms of the leading-order solid displacement $\boldsymbol{u}^{(0)}$, for a nematic liquid crystal flowing with velocity $\boldsymbol{v}^{(0)}=\dot{\boldsymbol{u}}^{(0)}$. The constitutive equation features the classical viscoelastic kinematic descriptors, that is the strain and strain rate, $\textsf{D}_{ \boldsymbol{x}}(\boldsymbol{u} ^{(0)})$ and $\textsf{D}_{ \boldsymbol{x}}(\boldsymbol{v} ^{(0)})$, respectively. However, despite the model being viscoelastic in nature, it shares similarities with a typical poroelastic model in that (a) the fluid pressure features in the constitutive relationship (with the associated incompressibility constraint (68b)) and is multiplied by the same tensor of coefficients that would appear in classical poroelastic formulations derived from the microstructure [14, 55], and (b) the effective stiffness tensor that is associated with the macroscale strains and given by $\left\langle {\mathbb {C}} {\mathbb {L}}+{\mathbb {C}} \right\rangle _{\textrm{s}}$ is indeed the drained elasticity tensor that would arise when deriving Biot’s equations via asymptotic homogenisation, as a consequence of the analogy between cell problems that we have highlighted in Section 5.3.1. The homogenised constitutive relationship shows that the contribution due to the nematic liquid crystal flow is encoded in both the modified viscosity tensor, through ${\mathbb {T}}( \boldsymbol{m}^{(0)})$, which depends on the nematic director $\textbf{m}^{(0)}$, as well as the auxiliary variable ${\mathbb {N}}$, which is likewise driven by the liquid crystal constitutive behaviour and hence the nematic director through ${\mathbb {T}}(\boldsymbol{m}^{(0)})$, cf. problem (60a–60b).

The homogenised model also features a volume load, $\tilde{\boldsymbol{h}}$, which again depends on the director through the interplay between macroscopic variations of the nematic liquid crystal, which are encoded in the fourth-rank tensor ${\mathbb {Q}}$, which depends on gradient of the director, and the geometric configuration which plays a crucial role in determining the solution of the auxiliary variables which appear in $\tilde{\boldsymbol{g}}$ (71) through explicit dependences on ${\mathbb {T}}$ and ${\mathbb {Q}}$ and through the auxiliary variables $\boldsymbol{\textsf{A}},\, {\mathbb {B}},\, \textsf{S}$. Finally, the nematic interaction with the solid phase enters through the cell-averaged quantity $\left\langle {\mathbb {C}}:\mathtt {\Pi }\right\rangle$.

Importantly,when ignoring the contribution due to the nematic elastic energy, that is, setting $\bar{K}=0$, we see that $\texttt{S}=0$, and thus, the auxiliary variables $\boldsymbol{\textsf{A}},\, {\mathbb {B}},\, \mathtt {\Pi }$ all vanish, leading to $\tilde{\boldsymbol{h}}=0$, showing that the nematic elasticity is driving the volume load at a macroscopic scale. Additionally, for a uniform nematic director, for which $\nabla _{\boldsymbol{x}} \boldsymbol{m}^{(0)}=0$, we have ${\mathbb {Q}}=0$ and therefore $\tilde{\boldsymbol{h}}=0$, and again there is no volume load at a macroscopic scale.

### A special case of the resulting homogenised model

The case in which the solid stresses contribution in the bulk can be neglected, i.e. $\nabla _{\boldsymbol{x}} \cdot \left\langle \mathsf { \sigma }_{\textrm{s}}^{(0)} \right\rangle _{{\textrm{s}}}\approx 0$, leads to the following simplified expression of our macroscopic governing equations:

72a $$\begin{aligned} \nabla _{ \boldsymbol{x}} \cdot \mathsf {\sigma }_{\text {Eff}} + \tilde{\boldsymbol{g}}&=0 \quad  &   \text { in } \ \Omega _{\textrm{f}}, \end{aligned}$$

72b $$\begin{aligned} \nabla _{ \boldsymbol{x}} \cdot \boldsymbol{v}^{(0)}&=0\quad  &   \text { in } \ \Omega _{\textrm{f}}, \end{aligned}$$

with the effective stress now defined as

73 $$\begin{aligned} \mathsf {\sigma }_{\text {Eff}}=\phi _\textrm{f} \bigg ( {\mathbb {T}}( \boldsymbol{m}^{(0)}):\textsf{D}_{ \boldsymbol{x}}\left( \boldsymbol{v} ^{(0)}\right) -\tilde{p} \textsf{I}\bigg ), \end{aligned}$$

and $\tilde{\boldsymbol{g}}$ given by (71). In this case, the problem reads as a differential problem for the leading-order velocity $\boldsymbol{v}^{(0)}$ and macroscale pressure $\tilde{p}$ (with associated incompressiblity constraint), which is to be supplemented by appropriate macroscale boundary conditions on $\partial \Omega _M$. We notice that the resulting governing equations read as an anisotropic Stokes-type problem where the viscosity tensor is depending on the leading-order nematic director $\textbf{m}^{(0)}$. In addition, the role of the spatial variations of the nematic director is also taken into account as they appear in the volume load $\tilde{\boldsymbol{g}}$ and contain contributions that are computed by solving the periodic cell problems (47a–47c), which depends on the specific microstructure geometry at hand as well as the nematic elastic energy and leading-order director $\boldsymbol{m}^{(0)}$.

## Concluding remarks

This study presents a mathematical model that describes the homogenised behaviour of a system comprising a linear elastic porous medium interacting with an incompressible nematic liquid crystal in the absence of inertia and body forces. The model uses a simplification of the Ericksen–Leslie theory in which the anisotropy of the nematic liquid crystal affects the fluid stress tensor, but the latter is still symmetric. The nematic director is then solely determined by the nematic elastic energy and governed by a system of PDEs, which does not depend on the fluid flow, while the fluid flow retains a dependency on the director configuration. We have then applied the asymptotic homogenisation technique to derive a macroscale set of governing equations, which describes the mechanical behaviour of the whole system. We have obtained a new model which represents the first example of upscaling of a differential problem via homogenisation involving an anisotropic fluid characterised by a constitutive equation, which explicitly accounts for the role of the nematic director and elastic energy. In particular, the model features macroscale coefficients and a volume load, which represent the clear link between the properties of the microstructure (in terms of the interplay between geometry, elastic stiffness, viscosities, and the nematic director) and the macroscale response of the material, as they are to be computed by solving microscale cell problems and discussed in Section 5.

The new model formally reads as a macroscale linear momentum balance for a material characterised by an effective viscoelastic-type response, supplemented by a volume load. The effective stress tensor given by relationship (69) reads as an additive decomposition with a poroelastic contribution and a purely viscous contribution. The poroelastic contribution includes the role of both the fluid pressure and the macroscale linearised strains, which are weighted by effective coefficients equivalent to the drained stiffness tensor and the Biot tensor of coefficients obtained when deriving classical poroelasticity via asymptotic homogenisation [14, 25, 37, 55]. The purely viscous contribution accounts for the role of the strain rate, with coefficients which depend on (a) the constitutive behaviour of fluid, hence the Leslie viscosities and the nematic director, (b) the stiffness of the solid phase, and (c) the geometry of the microstructure, which is encoded in appropriate auxiliary variables to be computed by solving microscale periodic cell problems. Therefore, while the model can be considered of viscoelastic-type, as it involves both strains and strain rates, it is not simply a standard Kelvin–Voigt model, as it also features the role of the pressure as a macroscale variable, with the associated macroscale incompressibility constraint (68b). The model could therefore be termed an *anisotropic poro-viscoelastic model*. The volume load (70) is directly related to the liquid crystal character of the fluid under consideration. It depends on the interplay between the microscale geometry, encoded once again in appropriate auxiliary variable to be computed on both the fluid and solid portions of the periodic cell, the material properties of the individual constituents (i.e. the fluid viscosities, the nematic director, and the stiffness of the elastic solid), and the nematic elastic energy. In particular, as we have remarked in Section 6, that the volume load reduces to zero either when ignoring the contribution due to the elastic energy, that is, for $\bar{K}=0$, or when considering a uniform nematic director, as in this case $\nabla _{\boldsymbol{x}} \boldsymbol{m}^{(0)}=0$ which implies ${\mathbb {Q}}=0$ and therefore $\tilde{\boldsymbol{h}}=0$.

We have derived the model under a number of simplifying assumptions, which can be relaxed in future works paving the way for several further developments. For instance, we have ignored the influence of external body forces, which could be related to the application of electro-magnetic fields to the system. Since liquid crystals responding to electro-magnetic fields, specifically through the reorientation of the director, an extension of this modelling framework could explore the potential of the model towards its practical applicability, with an electro-magnetic field inducing changes to the macroscale characteristics of the flow through the porous medium. This generalisation would entail a modification affecting both the governing equations for the nematic director, see, e.g. [68], and the balance of linear momentum in the fluid and solid domains, see, e.g. [1, 56] and [53] for recent example of application of the asymptotic homogenisation to the action of inhomogeneous volume loads in the context of fluid and solid mechanics problems, respectively. The modelling framework developed here has also neglected the role of inertia, which could be relevant when investigating elastic waves in porous media [14], and be considered as a weak coupling between the solid and fluid domains, as per Remark 3. The latter assumption means that the coefficients of the model are ultimately to be computed on either the solid or the fluid portion of the domain only, i.e. the fluid and solid problems decouple at the microscale, thus reducing computational complexity. This decoupling also happens in the context of classical poroelasticity when considering the fluid structure interaction between a linear elastic solid and a Newtonian fluid under the assumption of a characteristic parabolic fluid profile at the pore-scale [14, 52]. This is not the regime adopted in this work, but could be considered for situations where the resulting liquid crystal profile is not expected to dramatically differ from that of a Newtonian fluid.

However, whenever a strong coupling is to be considered, the problem can be upscaled by following the methodology reported, for example, in [46], so that we are expecting a coupling between the solid and fluid problems at the microscale, and strategies such as the one investigated in [31] can be utilised to treat linearised inertia terms and avoid a frequency domain representation of the fields. We further note that our modelling framework considers a symmetric stress tensor, thus avoiding the coupling from linear to angular momentum, although the coupling from angular to linear momentum is retained. A more general constitutive relationship for a nematic liquid crystal, for instance the full Ericksen–Leslie theory, would involve non-symmetric contributions to the fluid stress tensor that would lead to a fully coupled formulation accounting for the mutual influence of the fluid flow and the nematic director [69]. The next natural step for this work consists of the implementation of numerical simulations to solve the model, potentially exploring the possible real-world applications of nematic flow within a porous medium.

## Data Availability

No datasets were generated or analysed during the current study.

## Appendix. A multiscale model for the nematic director

We start from the simplest model governing the configuration of the nematic director in absence of external forces, namely a system of non-dimensional partial differential equations derived from the constrained minimisation of the Oseen–Frank elastic energy, under the one elastic constant approximation [69],

74a $$\begin{aligned} - \nabla ^2 \boldsymbol{m}+\lambda \boldsymbol{m}&=0 \quad  &   \text{ in } \ \tilde{\Omega }_{\textrm{f}}, \end{aligned}$$

74b $$\begin{aligned} (\nabla \boldsymbol{m}) \boldsymbol{n}&=0 \quad  &   \text{ on } \ \tilde{\Gamma }, \end{aligned}$$

74c $$\begin{aligned} \Vert \boldsymbol{m}\Vert ^{2}&=1 \quad  &   \text{ in } \ \tilde{\Omega }_{\textrm{f}}, \end{aligned}$$

where $\lambda$ is the Lagrange multiplier associated with the constraint $\Vert \boldsymbol{m}\Vert ^{2}=1$, and the problem is to be closed by prescribing appropriate conditions on the external boundary, for instance by assuming that $\boldsymbol{m}= \boldsymbol{m}_\textrm{s}$ on the fluid external boundary, where $\boldsymbol{m}_\textrm{s}$ is a given unit vector. The above system of PDEs can be equivalently obtained by considering the conservation of angular momentum for a nematic liquid crystal with the symmetric stress tensor described in the main text, under the one elastic constant approximation and ignoring time variations of the nematic director.

### The asymptotic homogenisation method

In this section, we employ the asymptotic homogenisation technique illustrated in Section 4 to upscale the system (74a–74c). In particular, we apply the standard assumptions described by equations (14–16) in terms of spatial scale decoupling, transformation of differential operators, and power series expansions to the system (74a–74c), obtaining

75a $$\begin{aligned} -\epsilon ^2 \nabla _{\boldsymbol{x}}^2 \boldsymbol{m}^{\epsilon } - \nabla _{\boldsymbol{y}}^2 \boldsymbol{m}^{\epsilon } - \epsilon \nabla _{\boldsymbol{y}} \cdot \nabla _{\boldsymbol{x}} \boldsymbol{m}^{\epsilon } - \epsilon \nabla _{\boldsymbol{x}} \cdot \nabla _{\boldsymbol{y}} \boldsymbol{m}^{\epsilon } +\epsilon ^{2} \lambda ^{\epsilon } \boldsymbol{m}^{\epsilon }=0&\quad  &   \text{ in } \ {\Omega }_{\textrm{f}},\end{aligned}$$

75b $$\begin{aligned} \epsilon \left( \nabla _{\boldsymbol{x}} \boldsymbol{m}^{\epsilon } \right) \boldsymbol{n} + \left( \nabla _{\boldsymbol{y}} \boldsymbol{m}^{\epsilon } \right) \boldsymbol{n}=0&\quad  &   \text{ on } \ {\Gamma },\end{aligned}$$

75c $$\begin{aligned} \boldsymbol{y}\text {-periodicity}&\quad  &   \text{ on } \ \partial \Omega _{\textrm{f}} \setminus \Gamma , \end{aligned}$$

subject to the constraint

76 $$\begin{aligned} \Vert \boldsymbol{m}^{\epsilon }\Vert ^{2}=1 \quad \text{ in } \quad \Omega _{\textrm{f}}. \end{aligned}$$

The above system of PDEs is defined on the periodic cell, as shown in Figure 1 and discussed in Section 4, and under the assumption of macroscopic uniformity defined in Remark 2. The function $\boldsymbol{m}^{\epsilon }$ is $\boldsymbol{y}$-periodic and represents the power series representation of the nematic director in terms of both spatial variables, i.e.

77 $$\begin{aligned} \boldsymbol{m}^{\epsilon }(\boldsymbol{x}, \boldsymbol{y} )=\sum _{l=0}^{\infty }\boldsymbol{m}^{(l)}(\boldsymbol{x}, \boldsymbol{y}) \epsilon ^l=\boldsymbol{m}^{(0)}(\boldsymbol{x}, \boldsymbol{y})+\epsilon \boldsymbol{m}^{(1)}(\boldsymbol{x}, \boldsymbol{y})+\epsilon ^2 \boldsymbol{m}^{(2)}(\boldsymbol{x}, \boldsymbol{y} )+\ldots \end{aligned}$$

In the next section, we derive a macroscale system of PDEs for the leading-order nematic director $\boldsymbol{m}^{(0)}$.

### Derivation of the macroscale model

In this section, we equate the coefficients of powers of $\epsilon$ in (75a–76) for $\epsilon ^{(l)},\ l=0,1 \ldots$ to derive a closed-form differential problem for the leading-order nematic director $\boldsymbol{m}^{(0)}$.

$$\begin{aligned} \boxed {\epsilon ^0} \phantom {00000000000000000000000000000000000000000000000000000000000000000000000000000000000000} \end{aligned}$$

Equating coefficients of $\epsilon ^0$ in the system (75a–75b) with constraint (76) we obtain:

78a $$\begin{aligned} -\nabla _{\boldsymbol{y}}^2 \boldsymbol{m}^{(0)}=0&\quad  &   \text{ in } \ {\Omega }_{\textrm{f}},\end{aligned}$$

78b $$\begin{aligned} \left( \nabla _{\boldsymbol{y}} \boldsymbol{m}^{(0)}\right) \boldsymbol{n}=0&\quad  &   \text{ on } \ {\Gamma }, \end{aligned}$$

78c $$\begin{aligned} \boldsymbol{y}\text {-periodicity}&\quad  &   \text{ on } \ \partial \Omega _{\textrm{f}} \setminus \Gamma , \end{aligned}$$

subject to the constraint

79 $$\begin{aligned} \Vert \boldsymbol{m}^{(0)}\Vert ^{2}=1 \quad \text { in } \quad \Omega _{\textrm{f}}. \end{aligned}$$

The system (78a–79) admits a unique solution up to a unit norm $\boldsymbol{y}$-independent vector-valued function, that is

80 $$\begin{aligned} \boldsymbol{m}^{(0)}=\boldsymbol{m}^{(0)}(\boldsymbol{x}). \end{aligned}$$

$$\begin{aligned} \boxed {\epsilon ^1} \phantom {00000000000000000000000000000000000000000000000000000000000000000000000000000000000000} \end{aligned}$$

Equating coefficients of $\epsilon ^1$ in the system (75a–76) yields

81a $$\begin{aligned} -\nabla _{\boldsymbol{y}}^2 \boldsymbol{m}^{(1)}-\nabla _{\boldsymbol{y}} \cdot \left( \nabla _{\boldsymbol{x}} \boldsymbol{m}^{(0)}\right) -\nabla _{\boldsymbol{x}} \cdot \left( \nabla _{\boldsymbol{y}} \boldsymbol{m}^{(0)}\right) =0&\quad  &   \text{ in } \ {\Omega }_{\textrm{f}},\end{aligned}$$

81b $$\begin{aligned} \left( \nabla _{\boldsymbol{x}} \boldsymbol{m}^{(0)}\right) \boldsymbol{n}+\left( \nabla _{\boldsymbol{y}} \boldsymbol{m}^{(1)}\right) \boldsymbol{n}=0&\quad  &   \text{ on } \ {\Gamma }, \end{aligned}$$

81c $$\begin{aligned} \boldsymbol{y}\text {-periodicity}&\quad  &   \text{ on } \ \partial \Omega _{\textrm{f}} \setminus \Gamma , \end{aligned}$$

subject to the constraint

82 $$\begin{aligned} \Vert \boldsymbol{m}^{(0)}\Vert ^{2}=1 \quad \text { in } \quad \Omega _{\textrm{f}}. \end{aligned}$$

We observe that $\nabla _{\boldsymbol{y}} \cdot \left( \nabla _{\boldsymbol{x}}\boldsymbol{m}^{(0)}\right) =0$ and $\nabla _{\boldsymbol{x}} \cdot \left( \nabla _{\boldsymbol{y}} \boldsymbol{m}^{(0)}\right) =0$ because $\boldsymbol{m}^{(0)}$ depends only on $\boldsymbol{x}$. For this reason, the system (81a–81c) can be recast simply as:

83a $$\begin{aligned} -\nabla _{\boldsymbol{y}}^2 \boldsymbol{m}^{(1)}&=0&\quad&\text{ in } \ {\Omega }_{\textrm{f}},\end{aligned}$$

83b $$\begin{aligned} \left( \nabla _{\boldsymbol{y}} \boldsymbol{m}^{(1)}\right) \boldsymbol{n}&=-\left( \nabla _{\boldsymbol{x}} \boldsymbol{m}^{(0)}\right) \boldsymbol{n}&\quad&\text{ on } \ {\Gamma }, \end{aligned}$$

83c $$\begin{aligned}&\quad \ \ \boldsymbol{y}\text {-periodicity}  &   \text{ on } \ \partial \Omega _{\textrm{f}} \setminus \Gamma , \end{aligned}$$

The above problem reads as a periodic Laplace problem for the vector-valued function $\boldsymbol{m}^{(1)}(\boldsymbol{x}, \boldsymbol{y})$ and admits a unique solution up to an arbitrary $\boldsymbol{y}$-independent function, which we denote by $\boldsymbol{d}(\boldsymbol{x})$. By linearity and knowing that $\nabla _{\boldsymbol{x}} \boldsymbol{m}^{(0)}$ is $\boldsymbol{y}$-independent, we can state the following ansatz for $\boldsymbol{m}^{(1)}$:

84 $$\begin{aligned} \boldsymbol{m}^{(1)}(\boldsymbol{x}, \boldsymbol{y})= \mathcal {W}: \nabla _{\boldsymbol{x}} \boldsymbol{m}^{(0)}+\boldsymbol{d}(\boldsymbol{x}). \end{aligned}$$

The microscale gradient of the order one nematic director can also be rewritten as follows

85 $$\begin{aligned} \nabla _{ \boldsymbol{y}} \boldsymbol{m}^{(1)} = {\mathbb {G}}: \nabla _{ \boldsymbol{x}} \boldsymbol{m}^{(0)}, \end{aligned}$$

where the fourth-rank tensor ${\mathbb {G}}$ is defined by

86 $$\begin{aligned} {\mathbb {G}}:= \nabla _{\boldsymbol{y}} \mathcal {W}. \end{aligned}$$

The solution of the system (83a–83c) is therefore provided by the (84), with the third-rank tensor $\mathcal {W}$ given by the solution to the following cell problem:

87a $$\begin{aligned} -\nabla _{\boldsymbol{y}}^2 \mathcal {W}&=0&\quad&\text{ in } \ {\Omega }_{\textrm{f}},\end{aligned}$$

87b $$\begin{aligned} \left( \nabla _{\boldsymbol{y}} \mathcal {W}\right) \boldsymbol{n}&=-\textsf{I} \otimes \boldsymbol{n}&\quad&\text{ on } \ {\Gamma }, \end{aligned}$$

87c $$\begin{aligned}&\boldsymbol{y}\text {-periodicity}  &   \text { on } \ \partial \Omega _{\textrm{f}} \setminus \Gamma , \end{aligned}$$

In order to ensure uniqueness of the problem (which is necessary for instance to compute a numerical solution of the problem), we impose a further condition on the auxiliary third-rank tensor $\mathcal {W}$:

88 $$\begin{aligned} \langle \mathcal {W}\rangle _ {\textrm{f}}=0. \end{aligned}$$

The system (87a–87c) and the constraint (88) can be rewritten component-wise as:

89a $$\begin{aligned} \frac{\partial ^2 W_{ipq}}{\partial y_j \partial y_j}&=0&\quad&\text{ in } \ {\Omega }_{\textrm{f}},\end{aligned}$$

89b $$\begin{aligned} \frac{\partial W_{ipq}}{\partial y_j} n_j&= -\delta _{ip} n_q&\quad&\text{ on } \ {\Gamma }, \end{aligned}$$

89c $$\begin{aligned} \langle W_{ipq}\rangle&=0&\quad&\text{ in } \ \Omega _{\textrm{f}}, \end{aligned}$$

89d $$\begin{aligned}&W_{ipq}  &   \ \boldsymbol{y}\text {-periodic. } \end{aligned}$$

The problem (89a–89d) corresponds to a vector-valued Laplace problem for each couple of fixed indexes $(p,q)=1,..n$, where $n=2$ or 3.

$$\begin{aligned} \boxed {\epsilon ^2} \phantom {00000000000000000000000000000000000000000000000000000000000000000000000000000000000000} \end{aligned}$$

Finally, equating the coefficients of power $\epsilon ^2$ in the system (75a–75b) yields:

90a $$\begin{aligned} -\nabla _{\boldsymbol{x}}^2 \boldsymbol{m}^{(0)}-\nabla _{\boldsymbol{y}}^2 \boldsymbol{m}^{(2)}-\nabla _{\boldsymbol{x}} \cdot \nabla _{\boldsymbol{y}} \boldsymbol{m}^{(1)}-\nabla _{\boldsymbol{y}} \cdot \nabla _{\boldsymbol{x}} \boldsymbol{m}^{(1)} +\lambda ^{(0)} \boldsymbol{m}^{(0)}&=0 \quad  &   \text{ in } \ \Omega _{\textrm{f}}, \end{aligned}$$

90b $$\begin{aligned} \left( \nabla _{\boldsymbol{x}} \boldsymbol{m}^{(1)}\right) \boldsymbol{n}+ \left( \nabla _{\boldsymbol{y}} \boldsymbol{m}^{(2)}\right) \boldsymbol{n}&= 0 \quad  &   \text{ on } \ \Gamma . \end{aligned}$$

In order to finalise the derivation of the macroscale problem for the nematic director, we apply the cell average operator (18) to equation (90a), obtaining

91 $$\begin{aligned} -\int _{\Omega _{\textrm{f}}}\nabla _{\boldsymbol{y}}^2 \boldsymbol{m}^{(2)}d \boldsymbol{y}-\int _{\Omega _{\textrm{f}}}\nabla _{\boldsymbol{y}} \cdot \nabla _{\boldsymbol{x}} \boldsymbol{m}^{(1)}d \boldsymbol{y}-\int _{\Omega _{\textrm{f}}}\nabla _{\boldsymbol{x}}^2 \boldsymbol{m}^{(0)}d \boldsymbol{y}-\int _{\Omega _{\textrm{f}}}\nabla _{\boldsymbol{x}} \cdot \nabla _{\boldsymbol{y}} \boldsymbol{m}^{(1)}d \boldsymbol{y} +\int _{\Omega _{\textrm{f}}}\lambda ^{(0)} \boldsymbol{m}^{(0)}d \boldsymbol{y}=0. \end{aligned}$$

The divergence theorem can be applied to the first two integrals and we can use the macroscopic uniformity condition (19) to rearrange the third and fourth integrals so that we obtain

92 $$\begin{aligned} \begin{aligned} -\int _{\partial \Omega _{\textrm{f}} / \Gamma } \nabla _{\boldsymbol{y}} \boldsymbol{m}^{(2)} \cdot \boldsymbol{n}_{\Omega _{\textrm{f}}} \textrm{dS}-\int _{\partial \Omega _{\textrm{f}} / \Gamma } \nabla _{\boldsymbol{x}} \boldsymbol{m}^{(1)} \cdot \boldsymbol{n}_{\Omega _{\textrm{f}}} \textrm{dS}-\int _{\Gamma }\left( \nabla _{\boldsymbol{y}} \boldsymbol{m}^{(2)}+\nabla _{\boldsymbol{x}} \boldsymbol{m}^{(1)}\right) \cdot \boldsymbol{n}_{\Gamma } \textrm{d} S&\\ -\nabla _{\boldsymbol{x}} \cdot \int _{\Omega _{\textrm{f}}}\nabla _{\boldsymbol{x}} \boldsymbol{m}^{(0)}d \boldsymbol{y}-\nabla _{\boldsymbol{x}} \cdot \int _{\Omega _{\textrm{f}}}\nabla _{\boldsymbol{y}} \boldsymbol{m}^{(1)}d \boldsymbol{y} +\int _{\Omega _{\textrm{f}}}\lambda ^{(0)} \boldsymbol{m}^{(0)}d \boldsymbol{y}&=0 \end{aligned} \end{aligned}$$

where the vectors $\boldsymbol{n}_{\Omega _{\textrm{f}}}$ and $\boldsymbol{n}_{\Gamma }$ are the unit normals to $\partial \Omega _\textrm{f} / \Gamma$ and $\Gamma$, respectively. By taking into account $\boldsymbol{y}$-periodicity, the integrals over the periodic boundary $\partial \Omega _f \setminus \Gamma$ vanish, hence:

93 $$\begin{aligned} -\int _{\Gamma }\left( \nabla _{\boldsymbol{y}} \boldsymbol{m}^{(2)}+\nabla _{\boldsymbol{x}} \boldsymbol{m}^{(1)}\right) \cdot \boldsymbol{n}_{\Gamma } \textrm{d} S-\nabla _{\boldsymbol{x}} \cdot \int _{\Omega _{\textrm{f}}}\nabla _{\boldsymbol{x}} \boldsymbol{m}^{(0)}d \boldsymbol{y}-\nabla _{\boldsymbol{x}} \cdot \int _{\Omega _{\textrm{f}}}\nabla _{\boldsymbol{y}} \boldsymbol{m}^{(1)}d \boldsymbol{y} +\int _{\Omega _{\textrm{f}}}\lambda ^{(0)} \boldsymbol{m}^{(0)}d \boldsymbol{y}=0. \end{aligned}$$

By employing relationship (90b) to eliminate the first integral in (93), we are left with

94 $$\begin{aligned} -\nabla _{\boldsymbol{x}} \cdot \int _{\Omega _{\textrm{f}}}\nabla _{\boldsymbol{x}} \boldsymbol{m}^{(0)}d \boldsymbol{y}-\nabla _{\boldsymbol{x}} \cdot \int _{\Omega _{\textrm{f}}}\nabla _{\boldsymbol{y}} \boldsymbol{m}^{(1)}d \boldsymbol{y} +\int _{\Omega _{\textrm{f}}}\lambda ^{(0)} \boldsymbol{m}^{(0)}d \boldsymbol{y}=0 \end{aligned}$$

We can now substitute the relationship (85) into equation (94) and, by rearranging the terms, we obtain the problem that describes the behaviour of the leading-order nematic director $\boldsymbol{m}^{(0)}$ in the macroscale domain $\Omega _M$:

95 $$\begin{aligned} \nabla _{\boldsymbol{x}} \cdot \left( {\mathbb {A}}: \nabla _{\boldsymbol{x}} {\boldsymbol{m}}^{(0)}\right) =\tilde{\lambda }\boldsymbol{m}^{(0)} \quad \text{ in } \ \Omega _{M} \end{aligned}$$

with the constraint $\Vert \boldsymbol{m}^{(0)}\Vert ^{2}=1\text { in } \ \Omega _{M}$.

Here the fourth-rank tensor ${\mathbb {A}}$ is defined as:

96 $$\begin{aligned} {\mathbb {A}}=\left\langle {\mathbb {I}}+{\mathbb {G}}\right\rangle _{{\textrm{f}}}=\frac{1}{|\Omega |} \int _{\Omega _{\textrm{f}}} \left( {\mathbb {I}}+{\mathbb {G}} \right) d \boldsymbol{y}, \end{aligned}$$

and $\tilde{\lambda }=\left\langle \lambda ^{(0)}\right\rangle _{{\textrm{f}}}$ is the Lagrange multiplier associated with the constraint $\Vert \boldsymbol{m}^{(0)}\Vert ^{2}=1$. The problem is then closed by prescribing appropriate conditions on the external macroscale boundary, for instance by setting $\boldsymbol{m}^{(0)}=\boldsymbol{m}_s$ on $\partial \Omega _M$. The fourth-rank tensor ${\mathbb {A}}$ encodes the properties of the microstructure and is in general not proportional to the identity, so that the problem describing the behaviour of the nematic director is in general anisotropic. The degree of anisotropy depends on the specific geometry at hand and is governed by the auxiliary tensor ${\mathbb {G}}$.
